# Stability indicating high performance thin layer chromatography method development and validation for quantitative determination of tetracycline hydrochloride in tetracycline hydrochloride active pharmaceutical ingredient (API) and its dosage forms

**DOI:** 10.1186/s13065-024-01183-6

**Published:** 2024-04-24

**Authors:** Misganaw Gashaw, Thomas Layloff, Ariaya Hymete, Ayenew Ashenef

**Affiliations:** 1 Department of Pharmaceutical Chemistry and Pharmacognosy, School of Pharmacy, College of Health Sciences, Addis Ababa University, P.O. Box. 1176, Addis Ababa, Ethiopia; 2 Department of Pharmacy, College of Health Sciences, Debre Markos University, P.O. Box. 269, Debre Markos, Ethiopia; 3 Granite City, USA; 4 Center for Innovative Drug Development and Therapeutic Trials for Africa (CDT-Africa), College of Health Sciences, Addis Ababa University, Addis Ababa, Ethiopia

**Keywords:** Analytical method, High performance thin layer chromatography (HPTLC), Medicine quality, Tetracycline HCl

## Abstract

Simple, quick, cost-effective, and environmentally friendly analytical methods for quality assurance and control roles for different medicines, including Tetrcyclines, are most significantly needed. Also, different thin layer chromatography (TLC)-based methods for tetracycline identification exist, but high performance thin layer chromatography methods based on modern state- of- the art equipment are still nonexistent. Thus, in this study, analytical method development and verification were done by high performance thin layer chromatography (HPTLC) (using an automated equipment model) using glass plates coated with silica gel 60 F_254_ after treating with 10% Na_2_EDTA. Validation was carried out according to International Council for Harmonization (ICH) guidelines. A mobile phase formed from ethyl acetate, acetonitrile, methanol, and 1% aqueous ammonia in the composition of 4.4:19.6:10:6 volume to volume ratio (V/V) was used. R_f_ value, percentage recoveries, linearity ranges, limit of detection (LOD), and limit of quantitation (LOQ) for the developed HPTLC method were 0.28, 100.83–106.25%, 160–560 ng/band (r^2^ values of 0.9999), 31.9 ng/band, and 96.7 ng/band, respectively. The results of the sample assays conducted using the new method and the United States Pharmacopoeia (USP) high performance liquid chromatography (HPLC) method were 91.59% to 108.3% and 90.83% to 102.85%, respectively. The F test for the aforementioned methods was 2.01, which is smaller than the tabulated F value of 5.05 with 5 degrees of freedom at a 95% confidence range, proving that the newly developed HPTLC and HPLC pharmacopoeial methods can be used interchangeably.The newly developed HPTLC method is easy, economical, specific, accurate, and roboust, thus it can be employed in a range of settings that require quality control and assurance activities of tetracycline hydrochloride (TC-HCl) in bulk and ointment dosage forms.

**Supplementary Information:**

The online version contains supplementary material available at 10.1186/s13065-024-01183-6.

## Introduction

### Background

**Fig. 1:**
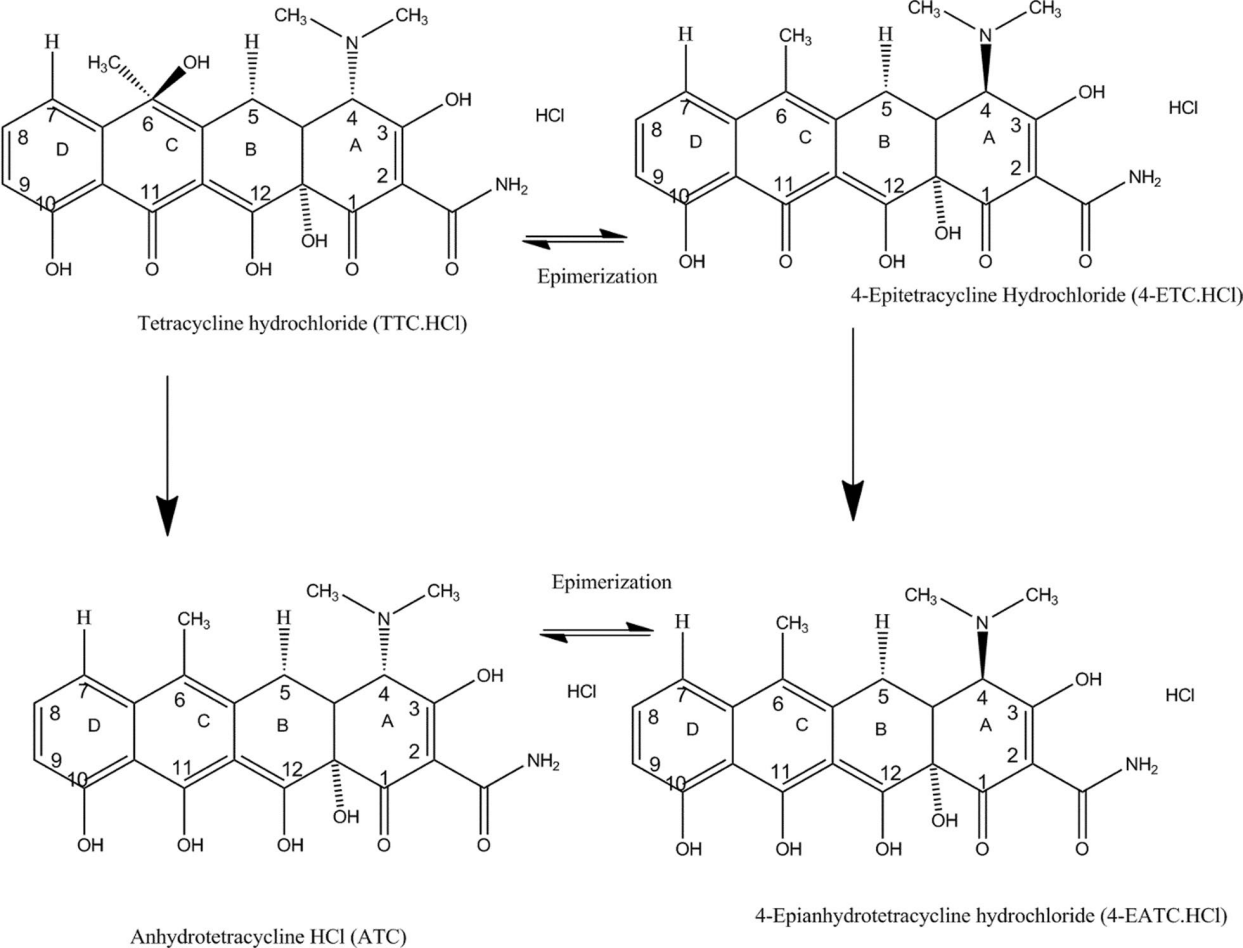
Chemical structures of tetracycline hydrochloride (TC-HCl), epitetracycline HCl (4-ETC), Anhydrotetracycline hydrochloride (4-ATC) and 4-Epi-anhydrotetracycline HCl (4-EATC)

Tetracyclines (TC) are a large family of antibiotics that were discovered in nature specifically from microorganisms. The directly extracted one among them is chlortetracycline, obtained from the *Streptomyces spp.* genus of *actinobacteria* [1]. Natural tetracyclines were used to create semi-synthetic or synthetic derivatives. Tetracycline HCl as a salt is prepared from naturally occurring chlorotetracycline by catalytic reduction [2]. It is a naphthacene antibiotic synthesized semi-synthetically from chlortetracycline (Fig. 1). It also had a chemical formula of C_22_H_24_N_2_O_8_.HCl, a molecular mass of 480.9 g/mol, a melting point of 417 ºF, 10 mg/mL solubility in methanol [3]. It is found as crystals or fine, bright yellow powder. The pH of tetracycline HCl solution (2% aqueous solution) is between 2.1 and 2.3 [4, 5].

The major impurities of tetracyclines occurring due to degradation, storage under adverse conditions, and other stress conditions can result not only in a loss of potency but also in the formation of toxic products [6]. Anhydrotetracycline (ATC), 4-epitetracyline (ETC), and 4-epianhydrotetracycline(EATC), are the major impurities. Anhydro tetracycline hydrochloride is chemically known by the IUPAC name 4S,4aS,12aS)-4-(Dimethylamino)-3,10,11,12a-tetrahydroxy-6-methyl-1,12-dioxo-1,4,4a,5,12,12a-hexahydrotetracene-2-carboxamide monohydrochloride). It had a molecular formula of C_22_H_22_N_2_O_7_·HCl and a molecular weight of 462.9 g/mol. It has to be stored in a refrigerator at 2–8 °C. Its solubility is ≥ 12.5 mg/mL in H_2_O; ≥ 15.43 mg/mL in EtOH with gentle warming; and ≥ 2.42 mg/mL in DMSO with ultrasonic. 4-epitetraycline with an IUPAC name of (4*R*,4*aS*,5*aS*,6*S*,12*aR*)-4-(dimethylamino)-1,6,10,11,12*a*-pentahydroxy-6-methyl-3,12-dioxo-4,4*a*,5,5*a*-tetrahydrotetracene-2-carboxamide; hydrochloride, molecular formula C_22_H_25_ClN_2_O_8,_ and molecular weight of 480.9 g/mol. It appears as a pale yellow to very dark yellow solid. Its solubility pattern is slightly soluble in DMSO, methanol, and water. It is light sensitive. It had to be stored below − 20 °C 4-Epianhydrotetracycline hydrochloride had an IUPAC name of (4R,4aS,12aS)-4-(Dimethylamino)-3,10,11,12a-tetrahydroxy-6-methyl-1,12-dioxo-1,4,4a,5,12,12a-hexahydrotetracene-2-carboxamide. Its molecular formula and weight are C_22_H_23_ClN_2_O_7_ and 462.88, respectively. Its appearance is dark orange to red solid. It is very slightly soluble in water and slightly soluble in methanol.It had to be stored at 2–8 °C in a refrigerator [7, 8]. Both chemical structures of the TTC. HCL and major impurities and how they are formed by epimerization or removal of water have been shown in Fig. 1.

A variety of microorganisms, such as gram-positive and gram-negative bacteria, chlamydia, mycoplasmas, rickettsiae, and protozoan parasites, are susceptible to tetracycline HCl. Tetracyclines have also been demonstrated to be effective against viruses. Despite the rise of resistance, tetracycline continues to be the treatment of choice for rickettsiae, chlamydial, trachoma, conjunctivitis, sinusitis, and other chlamydiaceae tracomatis infections. Tetracyclines are also very useful in veterinary medicine and, as such, are currently in wide use in the sector [9, 10].

Different dosage forms of tetracycline HCl include capsules, tablets, boluses, sachets, oral suspensions, injectable powders, ophthalmic ointments and drop solutions, skin ointments, and others. Different clinical conditions may be treated with tetracycline HCl eye and skin ointments. Tetracycline HCl 1% eye ointment is administered topically to the eyelid to treat superficial ocular infections. Various bacterial skin infections are treated and prevented with tetracycline HCl skin cream at a 3% concentration [11]. Chlamydial and gonococcal conjunctivitis in newborns and uncomplicated bacterial conjunctivitis are empirically treated with topical tetracycline HCl. In neonates, the drug should be applied to the conjunctiva as a thin strip in a single daily dose [10].

According to the World Health Organization’s Essential Medicines List (WHO EML), tetracycline hydrochloride 1% eye ointment can be used as an anti-infective agent [12]. Tetracycline eye ointment and 1% solution (eye drop) preparations are also in the WHO EML and are also mentioned in the Ethiopian medicine formulary for the treatment of superficial bacterial infections of the eye (purulent conjunctivitis), trachoma, and the prevention of gonococcal and nongonococcal ophthalmia neonatorum [13].

Various analytical methods have been documented to identify and assay tetracycline HCl in different formulations. The methods reported include microbiological assay [14], thin layer chromatography [15], UV–visible spectroscopy [16–19], flame atomic absorption spectroscopy, infrared spectroscopy [20], electroanalytical methods [21–23], and HPLC [20, 24, 25]. Official methods for identification and assay are based on infrared spectroscopy, UV–visible spectroscopy, paper chromatography, thin layer chromatography (TLC), and liquid chromatography (LC) techniques [26].

TLC based methods for tetracycylines had been reported previously [15, 27, 28]. The methods employ dichloromethane/methanol/water as the mobile phases. Detection is either by simple naked eye observation of spots or using charge coupled fluorescence densitometry and involves post-developmental treatment. Both of these methods are aimed at identification, and none of them are based on the current state-of-the art developed HPLC instruments capable of generating almost to equivalent analytical data to the golden standard methods for pharmaceutical analysis by pharmacopoeial methods such as—high performance liquid chromatography(HPLC)-based methods.

Though the aforementioned analytical techniques are available for the identification and quantification of tetracycline HCl in bulk and pharmaceutical dose forms, they do have some drawbacks that make it necessary to have an alternative. Specificity, sensitivity, and reproducibility limitations are associated with UV–visible spectroscopic analytical procedures to analyze tetracycline HCl [29]. HPLC is expensive, requires high skills for operation and maintenace, and is time-consuming [30]. Hence, an important issue is developing and validating an analytical technique that is reasonably easy to use, affordable, precise, quick, and environmentally friendly. Thus, this study is aimed at developing a novel new analytical method for TC HCl based on HPTLC analytical equipment.

## Materials and methods

### Materials

#### Equipments

Analytical balance (Scales Plus, USA), multifunctional heating oven (Binder GmbH, Germany), pH meter (Jenway, UK), hot plate with magnetic stirrer (Arex, Europe), sonicator (Bundlin Sonnorex super, Germany), LG refrigerator (LG Electronics, USA), and lab cold advanced freezer (Labcold, UK) were some of the instruments used. Glass tools were also used, including a glass mortar and pestle, different-sized class A graduated pipets (India), and different-sized class A volumetric flasks (Germany). Additionally, 100–1000 μL and 5–50 μL micro-pipettes (Dragon Lab, China) were used. For various tasks in this analytical work, different-sized beakers, conical flasks, glass and plastic funnels, measuring cylinders (England), and round amber glass bottles were also used.

#### Chemicals and reagents

For the forced degradation study, 30% (W/V) H_2_O_2_ solution (Park Scientific Limited, UK), 35.4% (W/V) HCl acid (Loba Chemie Pvt. Ltd., India), and Sodium Hydroxide pellet AR 98% (Sisco Research Laboratories Pvt. Ltd., India) were used. Other chemicals such as methanol AR 99% pure (Sisco Research Laboratories Pvt. Ltd., India); Acetone 99.5% AR/ACS (Loba Chemie Pvt. Ltd., India); Ethyl Acetate AR; acetonitrile AR; Ammonia solution 28%, chloroform AR (Carlo ERBA Reagents S.A.S, France); cyclohexane AR 99.5% pure (Park Scientific Limited, UK) were used for the preparation of the mobile phase system and the extraction solvent. Na_2_EDTA 99% AR /ACS (Loba chemie Pvt. Ltd., India) was used for preparation of 10% Na_2_EDTA solution, and then 40% NaOH (Oxford Lab Fine Chem, India) was used for 10% Na_2_EDTA solution adjustment to pH 9. The prepared solution was used for impregnating TLC and HPTLC plates. Ammonium oxalate (Avonchem, UK), dimethylformamide (Carlo ERBA Reagents S.A.S., France), and dibasic ammonium phosphate (Carlo ERBA Reagents S.A.S., France) were used for the analysis of drugs using the golden method (HPLC method) for the preparation of diluting solvents, sample solution preparation, and mobile phase preparation (Sigma-Aldrich, Germany).

#### Tetracycline HCl sample, standard, and impurities

Tetracycline HCl USP RS (Sigma, China) and USP RS standard impurities (Epitetracycline HCl (USA), Anhydrotetracycline HCl (India), and 4-Epi-anhydrotetracycline HCl (India)) were used in this work. All USP RS standards were kindly donated by the United States Pharmacopoeia, Maryland, USA. Tetracycline HCl BP working standard (98.24% potency) was kindly supplied by National Veterinary Institute (NVI) of Ethiopia. Three brands of tetracycline HCl 1% eye ointment preparations (Tetracycline HCl USP 1% eye ointment (Shanghai General Pharmaceutical Co Ltd, China), Galentic 1% eye ointment (Galentic Pharma Pvt. Ltd, India), Brassica 1% eye ointment (Brassica Pharma Pvt. Ltd, India)) and two brands of tetracycline HCl 3% skin ointment preparations (Galantic 3% skin oint (Galentic PharmaPvt.Ltd, India), Aurocycline 3% skin ointment (Aurochem Laboratories Pvt. Ltd, India)) and tetracycline HCl API (China), donated by NVI, Ethiopia, were used. Eye and skin ointments were purchased from pharmacy retail outlets in Addis Ababa, the capital city of Ethiopia. Further detailed information about standards and dosage forms has been given in the Additional file 1: Table S1.

#### Instrumentation and HPTLC condition

Preliminary tests to determine separation pattern, R_f_ value, and time taken for chromatography development were done on Merck silica gel 60 F_254_ precoated TLC plate 5 cm × 10 cm with 250 μm thickness with batch number HX302607 (Merck, Darmstaadt, Germany) in a 5 cm × 10 cm development chamber manually. The formed bands were overlooked with a UV-chamber (Camag, Muttenz, Switzerland) at 254 nm and 366 nm wavelength. After this, the semi-automated Camag HPTLC was used for further preliminary work. This instrument is equipped with a Linomat 5 sample applicator (Camag, Muttenz, Switzerland) for sample application using a 100 µl syringe (Hamilton-Bonaduz Schweiz, Camag, Switzerland). The chromatograms, which were developed in a glass twin-trough development chamber (Camag, Muttenz, Switzerland), were scanned with TLC scanner 4 (Camag, Muttenz, Switzerland). Camag’s WinCATS version 1.4.0 software (Camag, Muttenz, Switzerland) was used for different instruments' part order communication and data handling.

For the main work, a Camag HPTLC equipped with an Authomated Sample Applicator-4 (ATS 4) sample applicator (Camag, Muttenz, Switzerland) for sample application with a nitrogen stream supplied by a nitrogen tank automatically with a 25 µl syringe (Hamilton-Bonaduz Schweiz, Camag, Switzerland) was used. VisionCats version 3.1 software (Camag, Muttenz, Switzerland) was used to communicate between different parts of HPTLC and properly handle data. For taking images from a clean plate and after chromatogram development, TLC Visualizer 2 (Camag, Muttenz, Switzerland) was used. Ascending chromatogram development to a distance of 7 cm was performed in a presaturated and activated to 33% relative humidity with MgCl_2_ in a 20 cm × 10 cm Automated Development Chamber (ADC) 2 (Camag, Muttenz, Switzerland). The developed plates were dried for 2 min in ADC 2 with the presetted software command, and then densitometric scanning was done by the TLC scanner 4 (Camag, Muttenz, Swtzerland).

Merck silica gel 60 F_254_ precoated (250 μm thickness) 20 cm × 20 cm TLC plate with batch numbers: HX398477, HX86626259, HX99790429 which were cut into two equal parts to get 20 cm × 10 cm, and Merck silica gel 60 F_254_ precoated (200 μm thickness) 20 cm × 10 cm HPTLC plate with batch numbers: HX1850142, HX389048 (Merck, Darmstaadt, Germany) were used in the experiments. A saturation pad (Camag, Muttenz, Switzerland) was used for saturating development chambers. Camag TLC plate heater III (Camag, Muttenz, Switzerland) was used for activating the plate after derivatization [31].

The official method for assay [26] of tetracycline HCl was conducted by HPLC (Shimadzu, LC-2030 C 3D, Japan). It was equipped with a 280 nm detector, SPD 20A (Shimadzu, Japan). L_7_ guard column (5 µm-10 µm packing, 4.6-mm × 3-cm) for protection of main column from contaminants and C_18_ analytical column (4.6-mm × 25-cm) (Thermoscientific, England) were used. Lab solution software (Shimadzu, Japan) was used for instrument communication and data management. The aim was to compare the results of dosage form analysis performed by using the developed HPTLC method with the official HPLC method.

#### Softwares used

Data for method robustness analysis, i.e., Design of Experiments (DOE) and subsequent analysis of data was performed using Design-Expert (DE) version 13 (Stat-Ease Inc., Minneapolis, USA). Other statistical data were calculated by using Microsoft Excel 2013 (Microsoft Corporation, USA).

### Method development and optimization

#### Selection of solubilizing solvent

The solubility of drugs was estimated based on their physiochemical behavior. Some of the common solvents were evaluated for their availability, cost, suitability, and ability to dissolve analytes. The dosage form is in ointment formulation; thus, one of the considerations in solvent selection is the solvents’ ability to separate the API from the ointment base. Tetracycline HCl is fully soluble in polar solvents, and the ointment base could be dissolved in organic solvent. From different solvents, cyclohexane and methanol were selected to extract tetracycline HCl (TC-HCl) API from the ointment. After dissolving ointment with cyclohexane and methanol, the ointment base is in the cyclohexane layer and the TC-HCl API is in the methanol phase. Methanol was also used as a main and diluting solvent for proper solution formation [26].

#### Preparation of sample solution

The contents of five tubes of TC-HCl 3% skin ointment were bulked and mixed thoroughly. The same was done for 1% eye ointment. Portions of ointment equivalent to 300 mg and 100 mg TC-HCl for 3% skin ointment, and 1% eye ointment respectively, were accurately weighed and transferred into two different glass-stoppered conical flasks. 20 mL of cyclohexane R was added and shaken. Then, 35 mL of methanol were added and sonicated for 20 min. This mixture was filtered into a 100 mL volumetric flask, and the side of the conical flask was rinsed with 10 mL of methanol three times. Volume was adjusted to the mark with methanol to get 3 mg/mL and 1 mg/mL stock solutions for 3% skin ointment, and 1% eye ointment, respectively. A stock solution for API (1 mg/mL) was prepared using methanol as a solvent. Different concentrations required for the tests were prepared by diluting the stock solution. 0.06 mg/mL skin ointment and 0.05 mg/mL eye ointment working solutions were prepared by taking 4 mL from 3 mg/mL stock solution of skin ointment in a 200 mL volumetric flask and 5 mL of the 1 mg/mL eye ointment stock solution into a 100 mL volumetric flask, respectively. Stock and working solutions were stored at – 20 ºC and refrigerator (2–8 ºC) temperatures, respectively, in direct light exposure-protected environments [26].

#### Preparation of standard solution

Standard drug solution was prepared by dissolving accurately weighed TC-HCl with methanol solvent. All solutions were protected from light to avoid degradation. 0.05 mg/mL working solution was prepared by diluting stock solution using methanol solvent. The solutions of epitetracycline HCl (ETC-HCl), 4-epianhydrotetracycline HCl (4-EATC-HCl), and anhydrotetracycline HCl (ATC-HCl) were prepared with a 0.05 mg/mL concentration. A mixture of solutions of TC-HCl and the above three impurities was prepared by taking 5 mL of 1 mg/mL stock solution in a 100 mL volumetric flask and then adding 5 mg powder of each impurity. At the end, the solution was adjusted to volume by the solvent methanol [26].

#### Selection of mobile phase

The mobile phase for the HPTLC was selected from different solvents after multiple trials that took into consideration chemistry, previous TLC, HPLC literature [12, 27, 28], and the official TLC identification method [26, 33]. In mobile phase optimization, different concentrations were used. For the selection of the best solvent combination, the sharpness of the peak and the separation of the API from impurities were considered. The twin trough chamber was saturated by adding mobile phase and observing a saturation pad that was inserted in the other side of the twin chamber [32].

#### Selection of chromatographic condition

A sample was applied to 200.0 mm × 100.0 mm Merck HPTLC plates coated with silica gel 60 F_254_. Sample application was done at 8.0 mm away from the lower edge and 15 mm away from the plate's right and left side edges in the X axis. A sample was applied in the form of a band with a 6 mm length and a 10 mm gap between bands using the spray force of compressed N_2_ gas. The solvent was allowed to traverse 70.0 mm from the lower edge in an ascending manner. The filling speed of the sample application was 15 μL/s. The predosage volume and retraction volume were 200 nL individually. The dosage speed of the sample application was 150 nL/s. The solvent used for rinsing of the syringe was methanol. The nozzle of the syringe was not heated. Chromatogram development was done after ADC 2 was saturated for 20 min. The developed chromatogram was dried for 2 min. Data acquisition was done by image taking with TLC visualize 2 and densitometric scanning of chromatograms with TLC scanner 4 under a wavelength range of 260–410 nm against the developed chromatogram to estimate the wavelength of maximum absorption for the drugs of interest. From the spectrum, the better λmax for tetracycline HCl detection was confirmed [34].

#### Forced degradation studies

For hydrolysis based forced degradation studies, 5 mL of 1 mg/mL tetracycline HCl was added. 0.1 M HCl acidic and 0.1 M NaOH basic solutions were refluxed at 80 °C for 2 h. The refluxed mixtures were cooled and neutralized with 2.5 mL of 0.1 M NaOH basic and HCl acidic solutions at room temperature to stop further degradation. For oxidation based forced degradation, 5 mL of 1 mg/mL tetracycline HCl was mixed with 2.5 mL of 3% H_2_O_2_. The mixture was kept at room temperature for two hours before being heated to 80 °C for two minutes to prevent further oxidation. The volumes of all solutions were adjusted with methanol to a concentration of 0.1 mg/mL. To observe the pattern of impurity formation in different media, 4 μL of the degraded solution was applied to the HPTLC plate to get 400 ng/band. Tetracycline HCl powder was also kept for 6 h at 60 °C in an oven and at 254 nm in a UV irradiated chamber for heat and photon degradation studies, respectively. A stock solution was prepared by dissolving 10 mg in a 10 mL volumetric flask. Then, from this stock solution, a 0.1 mg/mL working solution was prepared [35].

### Method validation

The objective of the validation of an analytical method was to demonstrate the suitability of the developed method for its intended purpose. In our study, parameters such as accuracy, precision, specificity, detection limit, quantitation limit, linearity, range, robustness, and sample solution stability were assessed per standard methods as described by ICH, Food and Drug Administration of the US (FDA) guidelines [36–38]. Besides, the transferability of the developed method to another model of HPTLC equipment in another laboratory and the application of the newly developed method to real samples in comparison with the golden HPLC USP pharmacopoeial method were assessed.

### Quality assurance and quality control (QA/QC)

The laboratory experiments were performed in an ISO 17025 accredited laboratory following the standard operating procedures in place in the laboratory regarding quality assurance and quality control mechanisms of testing for medicines. Moreover, the experiments were performed per good laboratory practices and other recent relevant guidelines (e.g., ICH) stipulated in analytical method developments.

## Results and discussion

### Method development

#### Stationary phase employed

**Fig. 2:**
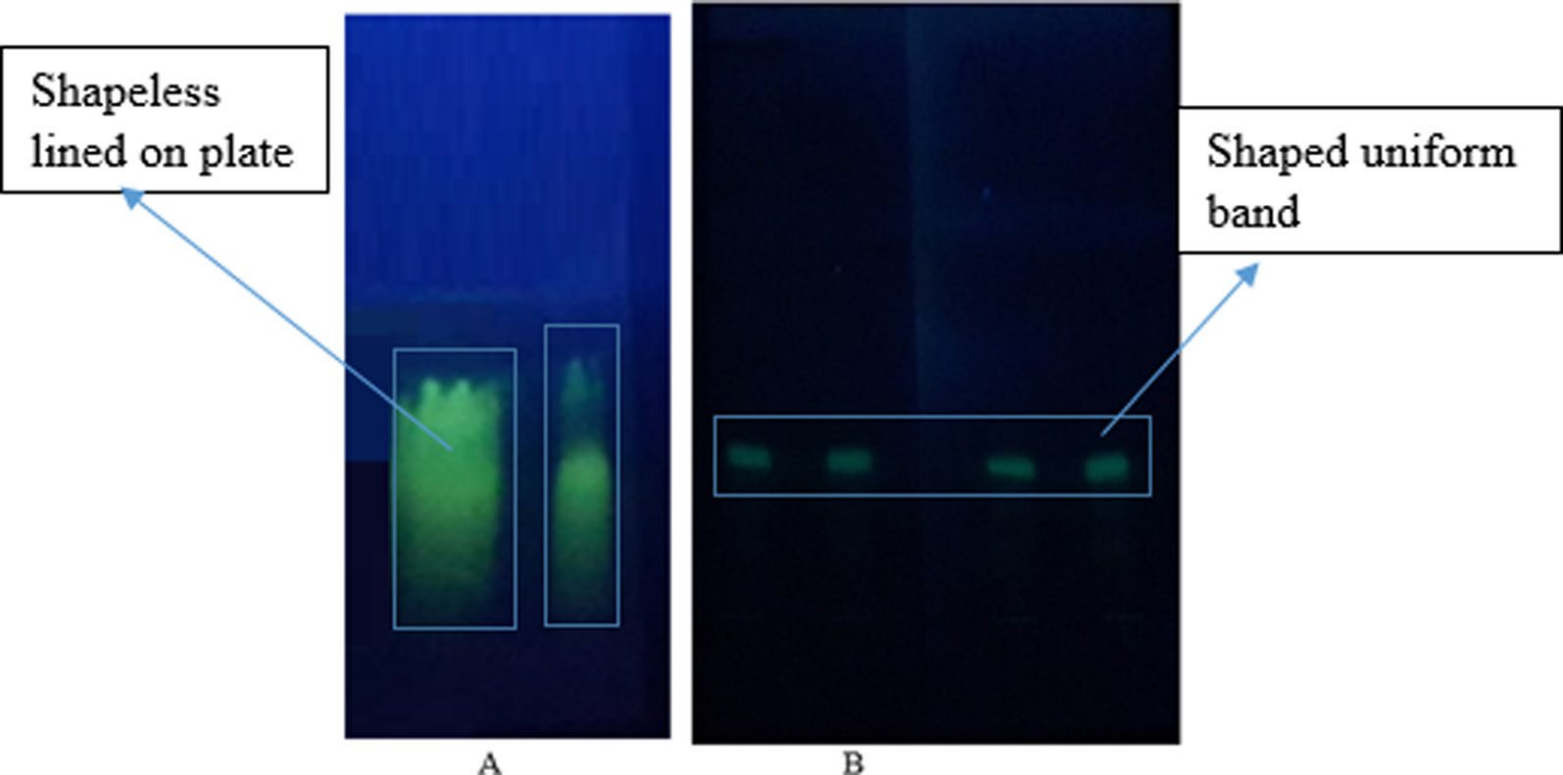
Chromatogram of TC-HCl on an untreated (**A**) and treated TLC plate (**B**)

The band formed on an untreated silica gel coated glass plate was not uniform; instead, it transformed into a shapeless line on the plate, causing band broadening (Fig. 2A).

The chromatograms of tetracycline HCl developed after treating the plate with 10% Na_2_EDTA at pH 9 were compacted, separated, and uniform (Fig. 2B). As a result, TLC and HPTLC plates precoated with silica gel 60F_254_ are chosen after being treated with 10% Na_2_EDTA, pH 9. Treatment of the TLC/HPTLC plates by Na_2_EDTA and NaOH in the analysis of tetracyclines had been reported previously [27, 28].

#### Mobile phase optimization

**Fig. 3:**
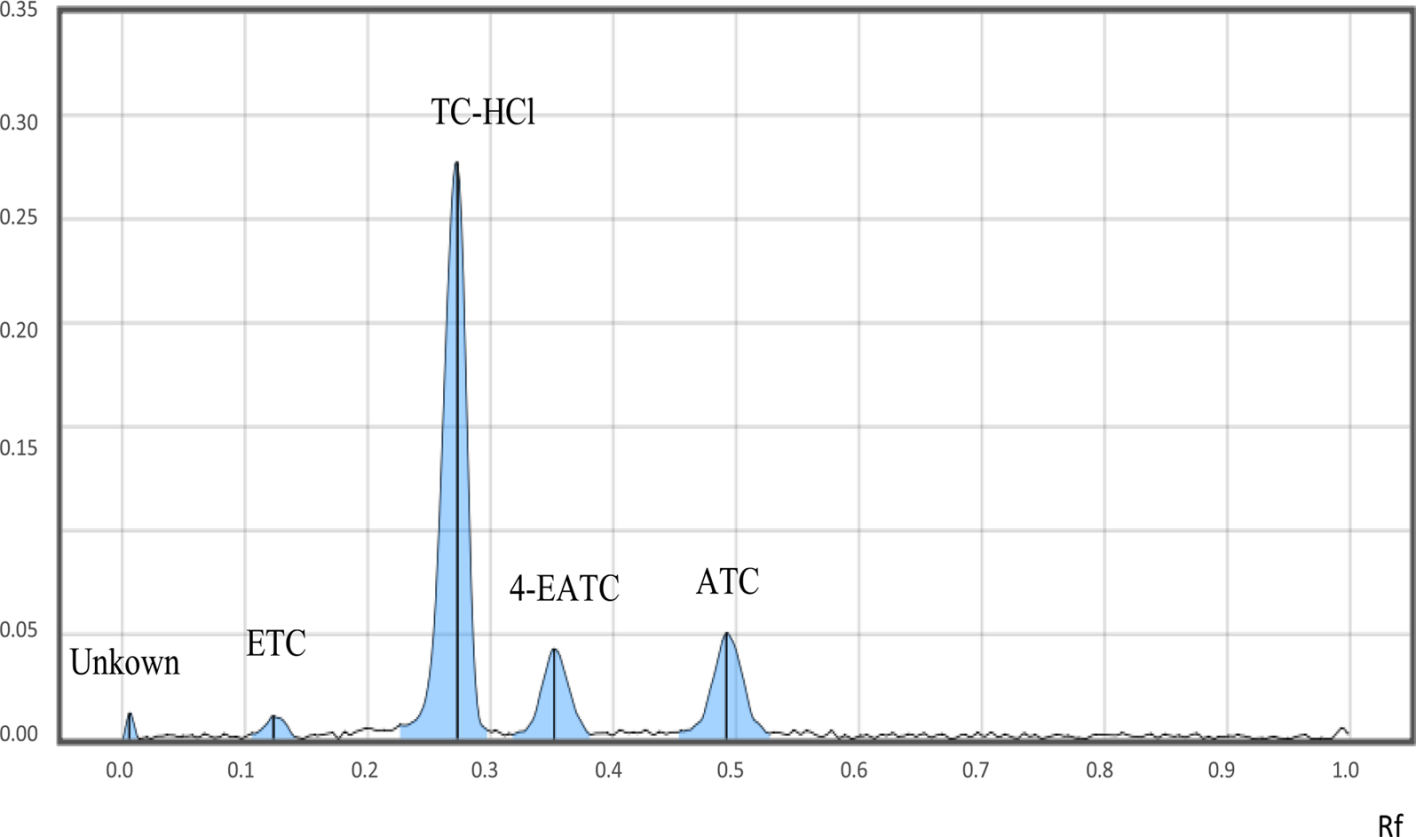
Peaks TC-HCl and its impurities (ETC, 4-EATC and ATC) scanned under TLC scanner 4 at 376 nm

After many trials, the mobile phase that worked well was formed by combining ethyl acetate, acetone, methanol, and 1% ammonia solution (4.4:19.6:10:6 (V/V)) (Table 1). The solution’s pH was 9.41. Under ADC 2 at 366 nm, the compacted bands and separated peaks for the analyte and impurities were seen in a well separated manner. Using the chosen mobile phase (MP) system, an analyte peak had good clarity and resolution. The optimal R_f_ value was generated with the selected MP system (Fig. 3). Additional information is mentioned in the Additional file 1: Figs.  S1–S3).

**Table 1:** Mobile phase compositions used in method optimization process

S.No.	Mobile phase composition	Proportions (v/v)	Observations
1	Acetone: EDTA: methanol: 1% ammonia solution	65:5:10:10	Poor resolution of peaks
2	Acetone: EDTA: methanol: 1% ammonia solution	65:5:10:15	Resolution problem between peaks
3	Chloroform: acetonitrile: methanol: 1% ammonia solution	11:49:25:15	Poor resolution between peaks
4	Chloroform: dimethylformamide: acetone: 1% ammonia solution	20:20:40:30	Longer development time, high noise
5	Ethyl acetate, acetone, methanol, and 1% ammonia solution^a,b^	4.4:19.6:10:6	Sharp peak with good resolution at R_f_ 0.27 $\pm$ 0.02
6	Ethyl acetate: acetonitrile: methanol: 1% ammonia solution	4.4:19.6:10:6	impurity peak overlap on analyte peak
7	Ethyl acetate: dimethylformamide: acetone: 1% ammonia solution	10:22:50:18	Peak resolution problem
8	Water: methanol: acetone: dichloromethane	10:70:20:60	Longer development time

^a^Selected mobile phase composition

^b^R_f_ for unknown impurity (0.005), ETC (0.123), TC-HCl (0.273), 4-EATC (0.352) and ATC (5, 0.492) with optimized mobile phase; resolution solution is the mixture of analyte and impurities

#### Spectrum scanning

The band of TC-HCl had maximum absorbance in the range of 370–382 nm with peak value at 376 nm. Thus, wave length of 376 nm was used for quantitative evaluation of TC-HCl (Additional file 1: Fig. S4).

#### Forced degradation studies

Even though there was decreasment of peak area of tetracycline HCl in all stress conditions (acid, base, oxidation, heat, photo), as it has been shown in Table 2, tetracycline HCl was completely degraded in 0.1 M HCl solution at 80 ºC for 2 h and in 3% H_2_O_2_. It was also highly degraded in 0.1 M NaOH. But its’ degradation in the oven, under UV chamber at 60 ºC and with 254 nm UV-light exposure, respectively, for 6 h was less in comparison with the others (Table 2). The new method’s ability to determine the drug in the presence of impurities formed under stress conditions has been shown in the Additional file 1: Figs. S5, S6).

**Table 2:** Peak area of 400 ng/band tetracycline HCl in stress conditions for forced degradation study

Condition	Formulation	Concetration	Tº and time	Peak area	Percent^a^ comparision
STD@	Solution	0.1 mg/mL	Room Tº	0.00684	–
Oven 60 ºC	Powder	50 mg	60ºC for 6 h	0.00665	97.2
UV chamber	Powder	50 mg	254 nm light for 6 h	0.00627	91.7
0.1 M NaOH	Solution	0.1 mg/mL	80 ºC for 2 h	0.00373	54.5
0.1 M HCl	Solution	0.1 mg/mL	80 °C for 2 h	0	0
3.0% H_2_O_2_	Solution	0.1 mg/mL	Room Tº for 2 h	0	0

@: The standard (STD) at room temperature

^a^Percent comparison is the peak area obtained in other conditions with that of the tandard room temperature condtion

### Method validation

#### Specificity

To test the specificity of the developed method, known standard impurities (ETC, 4-EATC, and ATC), with 0.05 mg/mL concentration, were introduced. TLC scanner 4 was used to perform densitometric scanning. TLC Visualizer II was used for image capture. Based on these data, the degree of separation between peaks and generated bands for analytes and impurities was assessed. Analyte and impurities were found to be well separated (Fig. 3). Furthermore, forced degradation was used to investigate specificity. Degradents were generated when the analyte was subjected to stress conditions (0.1 M HCl acid and 0.1 M NaOH base for hydrolysis, 3% H_2_O_2_ for oxidation, 60 ºC heat, and 254 nm light). There was a reduction in the peak area of the analytes. As a result, it can be said that the technique can distinguish between the analyte and contaminants created under stressful circumstances (Table 2).

#### Linearity and range

To examine the linear association between peak area and drug amount/band, six measurements were made in the range of; 160–560 ng/band (Table 3) using polynomial regression correlations. Peak area and amount of drug per band were correlated in the polynomial regression equation with a determination coefficient of 0.9999 (Table 3). The linearity of the current developed method (r^2^ = 0.9999) was observed to be better than that of the HPLC analytical method, which had a linearity range of 10–150 μg/mL and r^2^ of 0.9986 [40] and an r^2^ value of 0.999 at concentrations between 2.5 and 15 mg/mL [25] for the determination TC-HCl in an ointment of tetracycline HCl (Additional file 1: Fig. S7).

**Table 3:** Calibration parameters obtained using the HPTLC method

Parameter	HPTLC
Calibration range (ng/band	160–560
Coefficient of x^2^	− 7 × 10^–9^
Coefficient of x	2 × 10 ^−5^
Y-intercept	− 0.0005
Determination coefficient (R^2^)	0.9999
Correlation coefficient (r)	0.99995
Polynomial regression equation	Y = − 7 × 10^–8^ x^2^ + 2 × 10^–5^ x-0.0005

#### Accuracy

As shown in Table 4, the recovery of added standards into dosage form at 80%, 100%, and 120% was 106.3%, 101%, and 100.83%, respectively (Table 4). The average recovery for the developed HPTLC method at three levels was 102.7%, confirming its accuracy. The observed average recovery meets the percentage recovery acceptance criteria (98–102%) [38]. The following formula was used to calculate the percentage recovery:

**Table 4:** Recovery data at three concentration levels of TC-HCl

S.No.	Lable claim (ng/band)	Std added (ng/band)	(%) Std added	Total amnt (ng/band)	Recovered (ng/band)	Differ (ng/band)	Recover (%)
1	200	0	0	200	167	–	–
2	200	160	80	360	337	170	106.3
3	200	200	100	400	369	202	101.0
4	200	240	120	440	411	242	100.8
			Average recovery				102.7

$${\text{Recovery}}\,=\,(\frac{\mathrm{Analytical\,Result }}{\mathrm{True\,Value\,of\,Added\,STD}}){\text{X}}100\,{\%}$$

#### Precision

Repeatability and intermediate precision of the developed method were evaluated. For this purpose, nine determinations at three levels (160 ng/band, 200 ng/band, and 240 ng/band) were made. The average percent RSDs for intra-day and inter-day precisions were 0.9 and 1.19, respectively (Table 5). By applying and scanning 200 ng/band six times, the precision of the sample application and chromatogram scanning systems was validated. The method was confirmed to be acceptable in terms of sample application and scanning system precision, with percent RSDs of 1.56 and 0.65, respectively (Table 5). In general, the percent RSD for system and method precision was less than 2%, confirming that the established method was precise enough [38].

**Table 5:** Repeatability, intermediate precision and system precision test of tetracycline -HCl at 3 concentration (n = 3)

ng/band	HPTLC method					
	Intra-day precision			Intermediate precision		
	MPA	SD	%RSD	MPA	SD	%RSD
160	0.0024	2.8 × 10^–5^	1.2	0.0024	2 × 10^–5^	0.9
200	0.0035	1.7 × 10^–5^	0.5	0.0034	7 × 10^–5^	2.06
240	0.0043	4.5 × 10^–5^	1.0	0.0043	3 × 10^–5^	0.6
System precision test with 6 repeatation at 200 ng/band						

Peak area for sample application				PASS-peak area for sample scanning		
200	0.00276	4.3 × 10^–5^	1.56	0.00275	1.8 × 10^–5^	0.65

N.B.: MPA-mean peak area (n = 3), SD-standard deviation, RSD-relative standard deviation

$$\%{\text {RSD}} = 100(S/ \overline{x})\, {\text{(where S stands for standard deviation and}}\, \overline{x} \, \text{stands for mean}.$$

#### Limit of detection (LOD) and limit of quantification (LOQ)

Limit of detection and limit quantification for the developed method were calculated using standard deviations ($\upsigma$) of the calibration curve and the slope (S) of calibration curve. The slope was calculated from the calibration curve of the analysis, and similarly the standard deviations were driven from the standard deviations of the y-intercepts of regression lines. The formula used for calculation is: LOD = $3.3\upsigma$/S; LOQ = 10 σ/S [31]. The LOD and LOQ of the method were found to be 31.9 ng/band and 96.7 ng/band, respectively. The method had good sensitivity for analyzing API contents in pharmaceutical formulations.

#### Robustness

Based on the software (DE13), an ANOVA table was generated (Table 6) to describe the statistical model for the robustness study.

**Table 6:** Statistical parameters of ANOVA for the HPTLC method at 300 ng/band

S.No.	Parameters	Mobile phase composition		Different factors effect	
		Peak area	R_f_	Peak area	R_f_
1.	Model p-value	0.0747	0.1984	0.2019	0.1489
2.	Lack of fit	0.9594	0.8720	0.9627	0.891
3.	C.V. %	1.65	5.59	3.88	8.01
4.	R-squared	0.7506	0.8130	0.4210	0.7632
5.	Adj R-squared	0.5428	0.4857	0.2039	0.4789
6.	Pred R-squared	0.4968	0.4140	0.1069	0.4128
7.	Adeq precision	6.5380	6.0426	4.8219	6.1978
8.	Equation	Peak area = + 0.0054 − 0.0000A − 0.0000B + 0.0000C − 0.0001AB − 0.0001AC	R_f_ = 0.2898 − 0.015 A − 0.0105B + 0.0067C + 0.0077D + 0.005AB − 0.0077AC − 0.0067AD	Peak area = + 0.0051 + 0.0001B’ − 0.0001A’C’ + 0.0001A’D’	R_f_ = + 0.29 − 0.0075A’ − 0.042B’ − 0.005D’ − 0.0167A’B’ − 0.0075A’C’ − 0.015A’D’

^a^A-ethyl acetate volume, B-acetone volume, C-methanol volume, D-1% Ammonia solution volume, A’-mobile phase volume, B’- tank saturation time, C’-activation time, D’- solvent migration distance

The ANOVA results for the chosen Design of experiments (DOE) model for peak area and Rf of TC-HCl had demonstrated that the model's suitability for data analysis and investigation of the impact of factors on method robustness. Adequate precision, which must be greater than 4, measures the model's signal-to-noise ratio [41]. As shown in Table 6, adequate precision ranged from 4.8 to 6.5. These results indicated that the proposed models produced adequate signals. The value of the coefficient of variation (percent CV), which must be less than 10%, measures the model's reproducibility [41]. The % CV in this study was found to be in the range of 1.65–8.01 for all responses. The P value for lack of fit assesses model fitness for analysis using the proposed model [41]. A non-significant lack of fit is good, indicating that the model fits. The P-values for lack of fit in this study ranged from 0.8720 to 0.9627, indicating that the chosen model was appropriate and had no lack of fit. As shown in Table 6, the equation can be used to predict the response to a given level of each factor. By comparing the factor coefficients, the coded equation helps determine the factors' relative importance [41]. The model p values of the HPTLC method for each response are shown in Table 6 and are > 0.05, indicating that factors did not significantly affect the response. This indicates that the developed method was robust enough for its purpose.

**Fig. 4:**
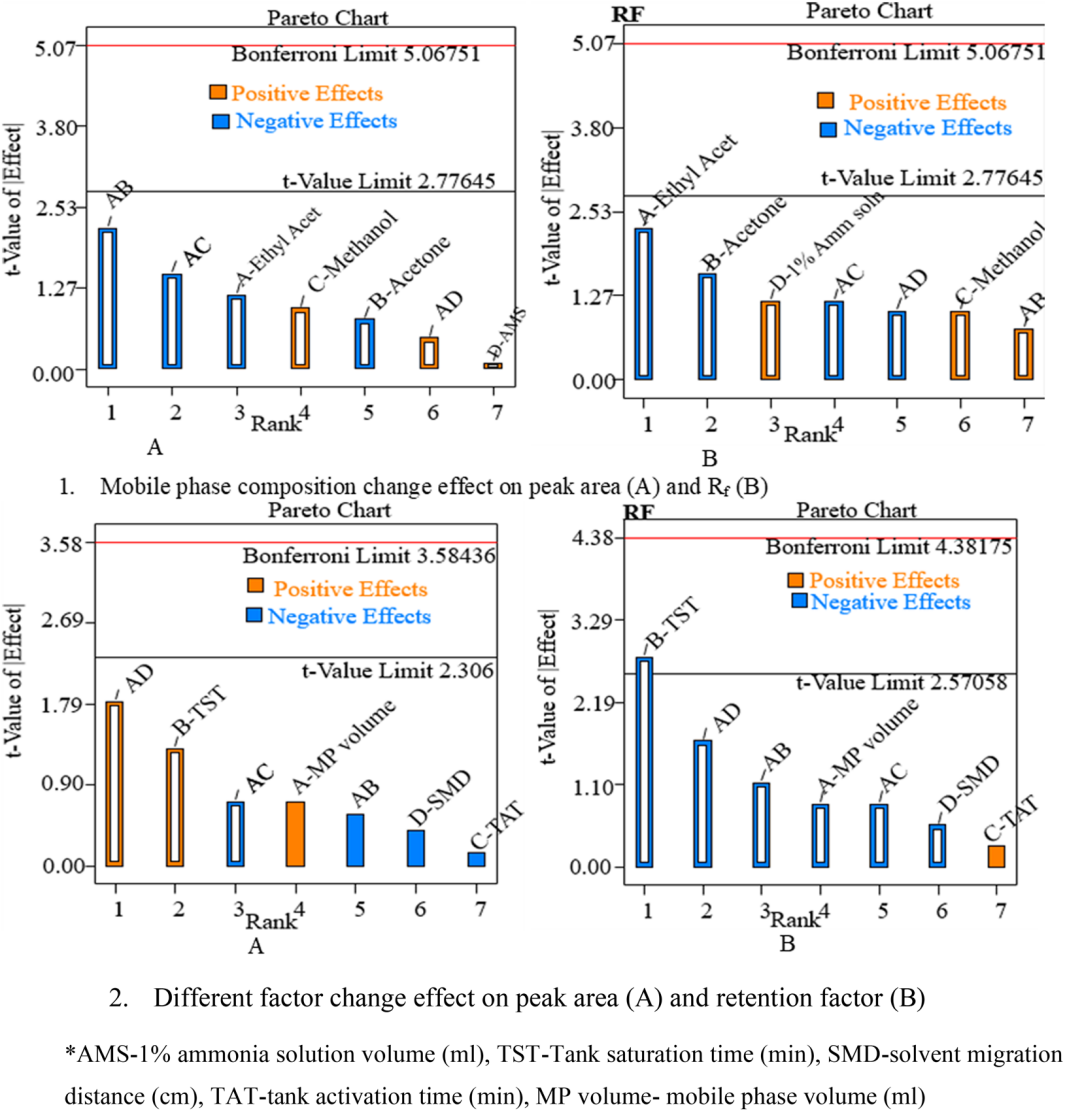
Pareto charts showing the effect of MP composition change(1) and change of other factors (mobile phase volume, saturation time, activation time and solvent migration distance) (2) on responses of the HPTLC method

**Fig. 5:**
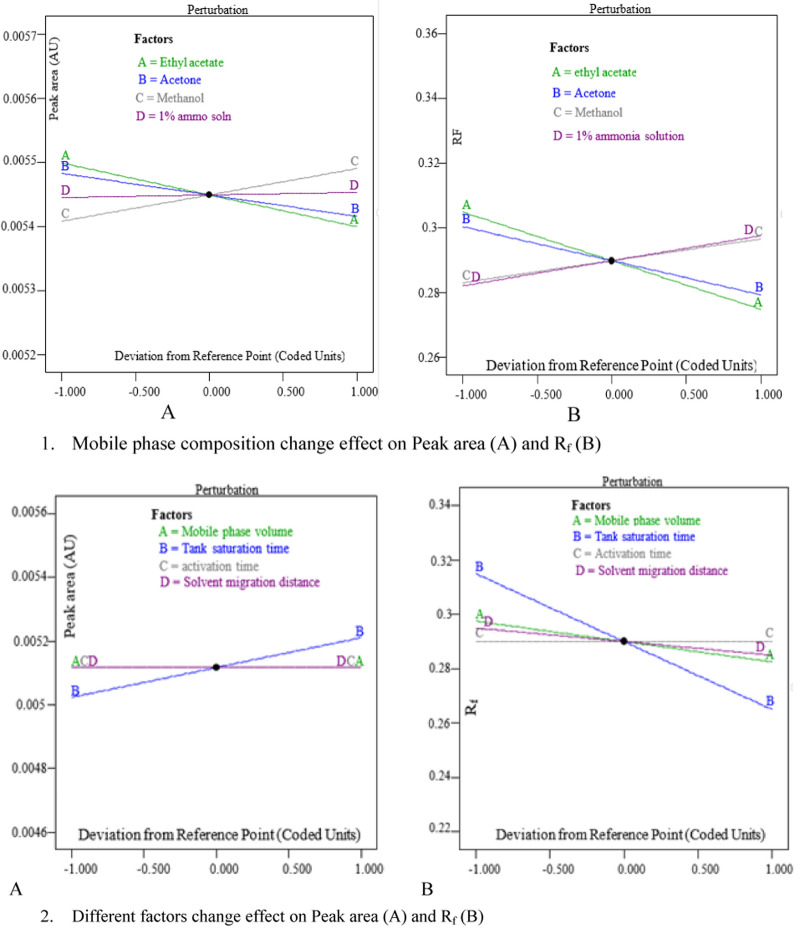
Perturbation plot for peak area response (1A&2A) and R_f_ response (1B&2B) for effect of factors on responses of HPTLC method

The DE 13 software's Pareto chart, perturbation plot, and 3D surface plot were also employed to assess the effect of the main factors and their interactions on the robustness of the developed method (Figs. 4, 5). The effect of varying the time gap between chromatogram development and scanning was investigated at 5, 10, 15, 20, 30, 40, 60, and 90 min (Table 7). As shown in Table 7, the developed method provided reproducible results up to 60 min after chromatogram development in a light protected environment. The amount of drug reduced was less than 5% up to 60 min. The method’s robustness was also assessed using the RSD of peak areas per band. It is not more than 2% up to 60 min [39].

**Table 7:** Time gap between development and scanning effect on peak area of 320 ng (n = 4)

Time gap	Mean peak area	SD	%RSD	Reduction	Reduction (%)
5 min	0.00569	6.96E−05	1.223936	0	–
10 min	0.00566	3.09E−05	0.546149	− 3E−05	− 0.52724077
20 min	0.00564	4.11E−05	0.728654	− 5E−05	− 0.87873462
30 min	0.005485	4.15E−05	0.757213	− 0.00021	− 3.60281195
40 min	0.005408	7.05E−05	1.303548	− 0.00028	− 4.96485062
60 min	0.005443	7.05E−05	1.295165	− 0.00025	− 4.34973638
90 min^a^	0.002323	4.49E−05	1.934575	− 0.00337	− 59.1827768

*Plate was exposed to light for 10 min

The Pareto chart was used to confirm the significance of factors. The factors with a bar line above the Bonferroni limit significantly affect the change in response as these factors change. Others with bar lines on the Pareto chart below the Bonferroni limit may also be significant. Under the t-value limit line, bar lines are unlikely to be significant for the change in response produced [41]. Because its bar line exceeds the Pareto chart's t-value limit (Fig. 4 (2B)), tank saturation time (B) on the retention factor of the drug may be significant. The other factor's bar lines were lower than the t-value. Changes in these variables, therefore, have little effect on the response of this HPTLC method. The developed method is thus sufficiently resistant to changes in factors in terms of retention factor and peak area.

The sensitivity of responses to factor changes was also demonstrated using perturbation plots. The factors with the steepest slopes show that even small changes in such factors can result in significant response changes [41]. Thus, such factors require special consideration. The slope of tank saturation time is the steepest of all, as shown in Fig. 5 (2B) indicating that tank saturation time has the greatest effect on the retention factor of the developed HPTLC method. However, its effect is only potentially significant, as it has been shown with a pareto chart (Fig. 4 (2B)).

As shown in the above figures of pareto charts, perturbation plots, and 3D surface plots in the Additional file 1: Figs S8, S9), the factors change in the given degree has no significant contribution to the change in responses. This implies that the developed method is resistant to small changes in the factors investigated in this robustness study. This indicates that the developed method is sufficiently robust.

#### Transferability of the method for semi-automated HPTLC

To check the applicability of the current method for semi-automated HPTLC, the sharpness and purity of peaks with the optimized method were assessed. At Rf of 0.31, there was a pure and sharp peak (Additional file 1: Fig. S16). The method was confirmed that to be specific enough for the determination of analytes in the presence of stressed formed impurities as well as in the intentionally added inherent impurity standards. The method using semi-automated HPTLC was linear in the range of 160 ng/band-560 ng/band with a determination coefficient (r^2^) of 0.9999 (Additional file 1: Fig. S18, Table S9). It was also accurate (average recovery, 99.0%), precise (intra-day precision (%RSD, 0.7–1.27), and inter-day precision (%RSD, 0.8–1.8)). The system precision of the method for sample application and chromatogram scanning was 1.99 and 1.56, respectively (Additional file 1: Table S12). The sensitivity of the method was with LOD (29.40 ng/band) and LOQ (89.08 ng/band). Hence, the developed method, with its given specificity, precision, accuracy, sensitivity, and robustness, can be used for analysis of TC-HCl in bulk and ointment dosage forms using a semi-automated HPTLC instrument. Details of data that show transferability are included as Additional file 1: Figs. S17–S21, Table S9–S14).

#### Sample stability study

Table 8 shows that the reduction in peak area of sample solution in 24 h after solution preparation was 7.69%, which is less than 10%. However, after 24 h, there was significant degradation, approaching and exceeding 10%. For example, at 48 h, the amount of reduction by degradation of solution was 12.82% (Table 8). To minimize systemic errors caused by sample solution degradation, drug analysis should be performed within 24 h of solution preparation in a light-protected environment.

**Table 8:** Stability study of 200 ng/band sample solution HPTLC method

S.No.	Time of analysis	MPA	Reduction (%)
	30 min	0.0039	–
	1 h	0.0039	–
	4 h	0.0038	− 2.56
	8 h	0.0037	− 5.13
	24 h	0.0036	− 7.69
	48 h	0.0034	− 12.82
	4 days	0.0018	− 52.63
	Time of analysis	MPA	Reduction (%)

^a^*MPA* mean peak area

#### Analysis of bulk substance and commercial dosage forms

Three brands of tetracycline HCl 1% eye ointments, two brands of tetracycline HCl 3% skin ointments, and the active pharmaceutical ingredient (API) tetracycline HCl were all analyzed in triplicates using the newly developed HPTLC method and also with the USP pharmacopoeial (HPLC) method. The API content was (108.3%) and (98.66%) for the HPTLC and HPLC methods, respectively. Tetracycline HCl 1% eye ointment and Tetracycline HCl 3% skin ointment were tested using dosage forms, and the results ranged from 91.59% to 104.25% and 93.03% to 103.74%, respectively (Table 9). These preparations were analyzed using the USP HPLC official method, and the results varied between 90.83% and 102.85% and 91.64% and 100.43%, respectively (Table 9).

**Table 9:** Assay of TC-HCl API and dosage forms with HPTLC and HPLC method

No. of assay	Content (%) of Tetracycline HCl in commercial form and API					
	TTC HCl API	Brassica eye ointment	Galentic eye ointment	TTC HCl USP	Galentic skin ointment	Aurocycline skin ointment
For HPTLC						
1	110.07	105.64	94.95	92.60	102.80	92.37
2	109.81	104.07	97.03	92.43	104.54	92.81
3	105.11	103.03	97.81	89.73	103.89	93.90
Average amount(%)	108.33	104.25	96.60	91.59	103.74	93.03
SD	2.28	1.31	1.48	1.61	0.88	0.78
%RSD	2.10	1.26	1.53	1.76	0.85	0.84
Mean ± SD	108.3 ± 2.28	104.5 ± 1.31	96.6 ± 1.48	91.59 ± 1.61	103.74 ± 0.88	93.03 ± 0.84
For HPLC						
1	98.58	102.46	96.30	90.92	100.42	92.47
2	98.61	102.42	96.31	91.32	100.26	92.45
3	98.78	103.68	96.24	90.23	100.62	90.01
Average amount (%)	98.66	102.85	96.28	90.83	100.43	91.64
SD	0.11	0.0.72	0.04	0.55	0.18	1.42
%RSD	0.11	0.70	0.04	0.60	0.18	1.54
Mean ± %RSD	98.66 ± 0.11	102.85 ± 0.72	96.28 ± 0.04	90.83 ± 0.55	100.43 ± 0.18	91.64 ± 1.42

Both methods had average API and dosage form contents that exceeded 90% of what was anticipated. Tetracycline HCl API results should not be less than 900 μg/mg, as per USP 2015. For dosage forms of skin and eye ointments, an acceptable specification range is between 90 and 125 percent. This indicated that the average API and dosage form content were within the acceptable range [26]. The tetracycline HCl assay results obtained using the recently developed HPTLC method and the USP official HPLC method were statistically compared using the F-ratio test. At a 95% confidence level for 5 degrees of freedom, the calculated F value was 2.01, which is lower than the reported F value of 5.05. (Table 10). This demonstrates that the current method and the accepted HPLC (USP) method have no statistically significant differences. The current technique can thus be used in place of the accepted HPLC technique.

**Table 10:** F-test two-sample for variances

Parameter	HPTLC	HPLC
Mean	99.62	96.78
Variance	46.51	23.15
Observations	6.00	6.00
Df	5.00	5.00
F	2.01	
P(F ≤ f) one-tail	0.23	
F Critical one-tail	5.05	

## Limitations of the study

The analytical method's optimum level was established using the one factor at a time (OFAT) method. In particular, a trial-and-error methodology was used to choose the solvents and optimize their proportion for the formation of the mobile phase. Furthermore, impurities formed during forced degradation were not identified. Future studies can work on the illucidation of the chemical structures of the impurities that are formed during forced degradation studies, including aspects of their quantitative, kinetic and mechanistic parameters.

## Conclusion

The findings of the present study demonstrated that tetracycline HCl could be assayed with good precision, accuracy, robustness, and specificity even in the presence of impurities using the newly developed and validated HPTLC method. The R_f_ value, percentage recoveries (accuracy), and linearity ranges, obtained for the developed HPTLC method were 0.28, 100.83–106.25%, 160–560 ng/band (r^2^ values of 0.9999) respectively. The limit of detection (LOD), and limit of quantitation (LOQ) found are also 31.9 ng/band, 96.7 ng/band, in the same order. The new method developed worked very well in compliance with the golden USP HPLC method for the analysis of real samples, i.e. dosage forms, as demonstrated by F-statistics (F calculated value of 2.01 versus F reported value of 5.05). The process is also easy, quick, inexpensive, and environmentally friendly. For quality assurance and control of tetracycline HCl in developing nations, it is thus a preferred analytical method.

### Supplementary Information

**Additional file 1: Table S1.** Standards and samples of TC-HCl with its impurities standards*. ***Table S2.** Mobile phase composition setting for robustness investigation corresponding to low (-), central (0) and high (+) level using fractional factorial design. **Table S3.** Different factors setting for robustness investigation corresponding to low (-), central (0) and high (+) level using fractional factorial design. **Figure S1.** Band of TC-HCl (0.26), EATC (0.274), ETC (0.11) and ATC (0.40) with ethyl acetate: acetonitrile: methanol: 1% ammonium solution (4.4:19.6:10:6 (V/V)) at 366nm of TLC visualizer II. **Figure S2.** Chromatogram of TC-HCl, 4-EATC, ETC and ATC observed under TLC visualizer II at 366 nm with optimised solvent system. **Figure S3.** Peaks for developed chromatograms with optimized mobile phase at 366nm under TLC Visualizer 2. **Figure S4.** UV spectrum of tetracycline HCl on scanning in the wave-length range of 260-410 nm. **Figure S5.** Bands of TC-HCl and products of its forced degradation with optimized mobile phase at 366 nm under TLC visualizer. **Figure S6.** Peaks for standard TC-HCl (1) and TC-HCl under stress conditions (0.1M NaOH (2), heated (3) and exposed to 254 nm (4)) for forced degradation study. **Figure S7.** Calibration curve of Tetracycline HCl using HPTLC method. **Table S4.** Execution of the fractional factorial experimental design for mobile phase composition and its responses to study robustness of the proposed HPTLC method. **Table S5.** Execution of the fractional factorial experimental design for different factors and its responses to study robustness of the proposed HPTLC method. **Figure S8.** 3D surface plot showing MP composition change effect on response of HPTLC method (peak area (1) & R_f_ value (2)) tetracycline HCl. **Figure S9.** 3D surface plot for effect of change of different factors on response of HPTLC method (peak area (1) & R_f_ (2)). **Table S6.** ANOVA analysis results for fractional factorial model of mobile phase composition change effect on HPTLC method responses. **Table S7.** ANOVA analysis results for fractional factorial model for different factors change effect on HPTLC responses. **Figure S10.** Peak of tetracycline HCl API and eye ointment pharmaceutical products with HPTLC method. **Figure S11.** Peak of TC-HCl working standard and skin ointment pharmaceutical products with HPTLC method. **Figure S12.** Peak of mixture of TC-HCl and 4-EATC (A) and TC- HCl WS using official HPLC method. **Figure S13.** Peak of TC-HCl skin ointment with official HPLC method. **Figure S14.** Peak of TC-HCl API and eye ointment with official HPLC method. **Figure S15.** Peak of TC-HCl eye ointment with USP HPLC method. **Figure S16.** Peak for tetracycline HCl (Rf, 0.30±0.02) with semi-automated HPTLC without background noise correction (A) and with background noise correction (B). **Figure S17.** Degradation of tetracycline HCl in 0.1M HCl at environmental T^O^ (1), 0.1M HCl at 80^O^c for 2hrs (2), 0.1M NaOH at environmental T^O^ (3), 0.1M NaOH at 80^O^c for 2hrs (4), 3% H_2_O_2_ solution (5) and standard solution (6). **Table S8.** Peak area of 400 ng/band TC-HCl in stress conditions using semi-automated HPTLC. **Figure 18.** Calibration curve of tetracycline HCl for semi-automated HPTLC. **Table S9.** Calibration parameters obtained from the semi-automated HPTLC method. **Figure 19.** Pareto charts of rank (X-axis) vs t-value of |effect| (Y-axis) showing the effect of MP composition change (1) and change of other factors (2) on responses of semi-automated HPTLC. **Table S11.** MP composition change effect on robustness of HPTLC-1 method with FFD. **Table S12.** Testing of robustness of factors change for semi-automated HPTLC method full factorial design. **Figure S20.** Perturbation plot for peak area response (1A&2A) R_F_ response (1B&2B) for effect of factors on responses HPTLC-1 method. **Table S13.** Statistical Parameters from ANOVA for semi-automated HPTLC. **Figure S21.** 3D surface plot showing effect of MP composition and different factors on HPTLC-1 response (1and 3–peak area. And 2 and 4–R_F_-value). **Table S14.** Stability study of 200 ng/band sample solution with semi-automated HPTLC method.

## Data Availability

Most of the data generated or analyzed during this study are included in this article. We have included additional data under the supplementary material.Moreover, queries can be directed to the corresponding author for any clarifications about the study if needed.
