# Supplementary material for: Stability indicating high performance thin layer chromatography method development and validation for quantitative determination of tetracycline hydrochloride in tetracycline hydrochloride active pharmaceutical ingredient (API) and its dosage forms
Source: BMC Chem. 2024 Apr 24;18(1):82. doi: 10.1186/s13065-024-01183-6 (PMC11040820; doi:10.1186/s13065-024-01183-6)
Supplement: Supplementary file 1 — Additional file 1: Table S1. Standards and samples of TC-HCl with its impurities standards. Table S2. Mobile phase composition setting for robustness investigation corresponding to low (-), central (0) and high (+) level using fractional factorial design. Table S3. Different factors setting for robustness investigation corresponding to low (-), central (0) and high (+) level using fractional factorial design. Figure S1. Band of TC-HCl (0.26), EATC (0.274), ETC (0.11) and ATC (0.40) with ethyl acetate: acetonitrile: methanol: 1% ammonium solution (4.4:19.6:10:6 (V/V)) at 366nm of TLC visualizer II. Figure S2. Chromatogram of TC-HCl, 4-EATC, ETC and ATC observed under TLC visualizer II at 366 nm with optimised solvent system. Figure S3. Peaks for developed chromatograms with optimized mobile phase at 366nm under TLC Visualizer 2. Figure S4. UV spectrum of tetracycline HCl on scanning in the wave-length range of 260-410 nm. Figure S5. Bands of TC-HCl and products of its forced degradation with optimized mobile phase at 366 nm under TLC visualizer. Figure S6. Peaks for standard TC-HCl (1) and TC-HCl under stress conditions (0.1M NaOH (2), heated (3) and exposed to 254 nm (4)) for forced degradation study. Figure S7. Calibration curve of Tetracycline HCl using HPTLC method. Table S4. Execution of the fractional factorial experimental design for mobile phase composition and its responses to study robustness of the proposed HPTLC method. Table S5. Execution of the fractional factorial experimental design for different factors and its responses to study robustness of the proposed HPTLC method. Figure S8. 3D surface plot showing MP composition change effect on response of HPTLC method (peak area (1) & Rf value (2)) tetracycline HCl. Figure S9. 3D surface plot for effect of change of different factors on response of HPTLC method (peak area (1) & Rf (2)). Table S6. ANOVA analysis results for fractional factorial model of mobile phase composition change effect on HPTLC method r [file 13065_2024_1183_MOESM1_ESM.docx]

Stability Indicating High Performance Thin Layer Chromatography (HPTLC) Method Development and Validation for Quantitative Determination of Tetracycline Hydrochloride in Tetracycline Hydrochloride Active Pharmaceutical Ingredient (API) and Its Dosage Forms

Misganaw Gashaw^1,2^, Thomas Layloff^3^, Ariaya Hymete^1,^ Ayenew Ashenef^1,4*^

^1^Department of Pharmaceutical Chemistry and Pharmacognosy, School of Pharmacy, College of Health Sciences, Addis Ababa University, P.O. Box. 1176, Addis Ababa, Ethiopia

^2^Department of Pharmacy, College of Health Sciences, Debre Markos University, P.O. Box. 269, Debre Markos, Ethiopia

^3^Consultant, USA, P O Box 286 Granite City, IL 62040-0286

^4^ Center for Innovative Drug Development and Therapeutic Trials for Africa (CDT-Africa), College of Health Sciences, Addis Ababa University, Ethiopia

Correspondence email: ayenew.ashenef@aau.edu.et

# MATERIALS AND METHODS

Table S1: Standards and samples of TC-HCl with its impurities standards

| S.N. | Brand | Batch | Man. date | Expiry date | Preparation |
| --- | --- | --- | --- | --- | --- |
| 1 | TC-HCl (97.1% potency) | R039W0 | * | 31/may/20 | Powder |
| 2 | ETC (97.8% potency) | R095C0 | * | current | Powder |
| 3 | ATC (95% potency) | R117XO | * | current | Powder |
| 4 | 4-EATC (94% potency) | R11400 | * | current | Powder |
| 5 | TC-HCl WS | WS/Tetr/20/06 | * | 29/04/22 | Powder |
| 6 | TC-HCl USP 1% | 201021 | 10/2020 | 10/2023 | eye oint |
| 7 | Galentic 1% | AF21134 | Sep.2021 | 10/.2024 | eye oint |
| 8 | Brassica 1% | A0L08 | 12/2020 | 11/2023 | eye oint |
| 9 | Galantic 3% | GF21007 | Jun.2021 | 05/2024 | Skin oint |
| 10 | Aurocycline 3% | (10) 6127 | 07/2021 | 06/2024 | Skin oint |
| 11 | TC-HCl API | * | * | 11/2022 | Powder |

*Not available, oint- ointment, TC-HCL-Tetracycline HCl, ATC-Anhydrotetracycline HCl, ETC-Epitetracycline HCl, 4-epianhydrotetracycline HCl, WS-Working standard, API-Active Pharmaceutical Ingredients

## Method validation

### Robustness

Table S2: Mobile phase composition setting for robustness investigation corresponding to low (-), central (0) and high (+) level using fractional factorial design

| Factors | Low level(-) | Zero level(0) | High level(+) |
| --- | --- | --- | --- |
| Ethyl acetate (mL) | 4.2 | 4.4 | 4.6 |
| Acetone (mL) | 19.4 | 19.6 | 19.8 |
| Methanol (mL) | 9.8 | 10 | 10.2 |
| 1% ammonia solution (mL) | 5.8 | 6 | 6.2 |

Table S3: Different factors setting for robustness investigation corresponding to low (-), central (0) and high (+) level using fractional factorial design

| Factors | Low level(-) | Zero level(0) | High level(+) |
| --- | --- | --- | --- |
| Mobile phase volume (mL) | 33 | 35 | 37 |
| Tanker saturation time(min) | 15 | 20 | 25 |
| Activation time (min) | 8 | 10 | 12 |
| Solvent migration distance (cm) | 6.8 | 7 | 7.2 |

# Results

## Method development

### Optimization of mobile phase

Different solvents and their relative proportions were tried for the selection of best mobile phase composition. Mobile phase formed from ethyl acetate: dimethylformamide: acetone: 1% ammonia solution with the proportion of 10:22:50:18 (V/V) was tested. Using this mobile phase, chromatogram development time was so long (40 min). When chloroform was used in the place of ethyl acetate chloroform: dimethylformamide: acetone: 1 % ammonium solution with the proportion of 20:20:40:30 (V/V), no change in the time required for chromatogram development. Water: methanol: acetone: dichloromethane of 10:70:20:60 (V/V) were also tried even though the time required for chromatogram development was not different from the previous composition. When methanol was used in the place of dimethylformamide and water, there was improvement in the duration of time required for chromatogram development but still there was resolution problem between peaks. There was not enough separation between impurities’ band and the analyte’s band using mobile phase formed from mixture of Acetone: EDTA: methanol: 1 % ammonia solution of 65:5:10:10 (65:5:10:15) (V/V)


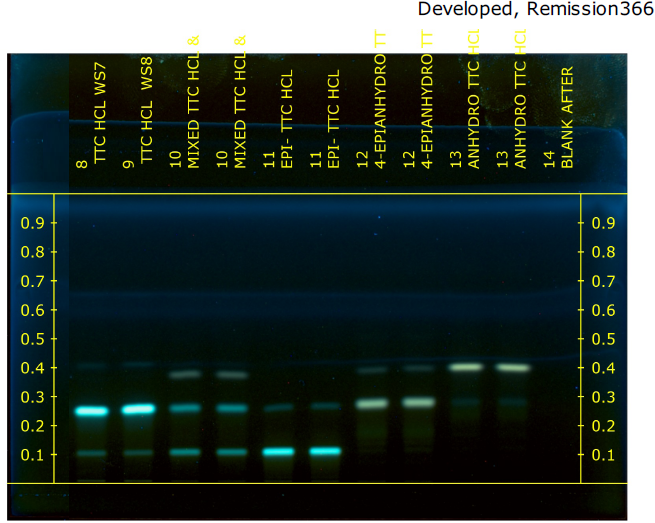


Figure S1: Band of TC-HCl (0.26), EATC (0.274), ETC (0.11) and ATC (0.40) with ethyl acetate: acetonitrile: methanol: 1% ammonium solution (4.4:19.6:10:6 (V/V)) at 366nm of TLC visualizer II

Other mobile phase (chloroform: acetonitrile: methanol: 1 % ammonium solution of 11:49:25:15 (V/V) were also attempted. As shown in Table 5, ethyl acetate: methanol: acetonitrile: 1 % ammonia solution with the proportion of 15: 22: 50: 18 (V/V) were mixed to get mobile phase. Resolution between peaks of analyte and impurities was highly improved even though it was not enough. Mobile phase formed from ethyl acetate: acetonitrile: methanol: 1% ammonium solution (4.4:19.6:10:6 (V/V)) was used for checking of tetracycline HCl band separation from the band of 4-epianhydrotetracycline HCl impurity. The band of this impurity and analyte were overlapped as it has been shown in Figure 1.

A mobile phase that worked well was formed by combining ethyl acetate, acetone, methanol, and 1% ammonia solution (4.4:19.6:10:6 (V/V)). The solution's p^H^ was 9.41. Under ADC 2 at 366 nm, the separated and compacted bands for the analyte and impurities were clearly well separated (Figure 2). Using the chosen mobile phase system, analyte peak was generated with good clarity and resolution (Figure 3). Under this system, the analyte R_f_ value was 0.27±0.02. As a result, the optimal system for this study was decided to be this mobile phase combination in the mentioned composition.


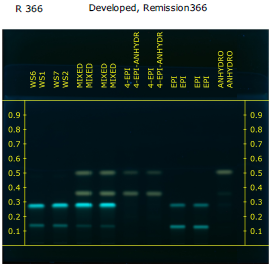


Figure S2: Chromatogram of TC-HCl, 4-EATC, ETC and ATC observed under TLC visualizer II at 366 nm with optimised solvent system


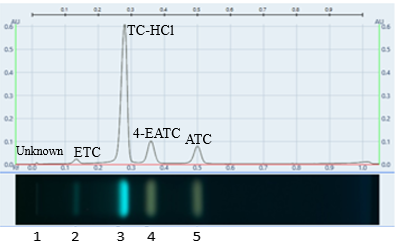


Figure 3: Peaks for developed chromatograms with optimized mobile phase at 366nm under TLC Visualizer 2

### Optimum wavelength

*
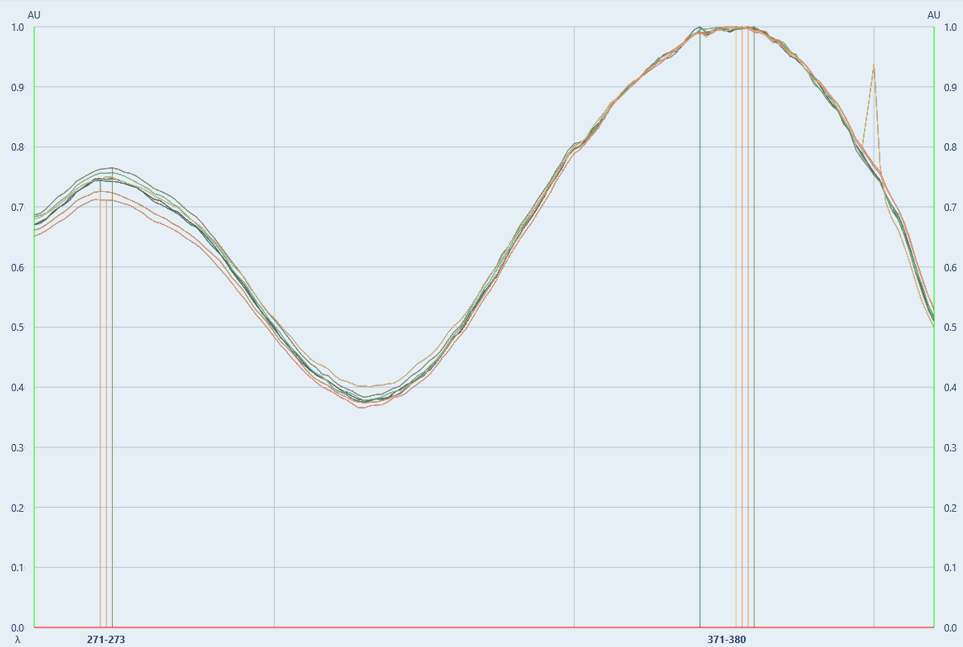
*The plate was scanned over the range of 260 nm to 410 nm wavelength to identify a wavelength that can give maximum absorbance response. The band had maximum absorbance in the range of 370 nm-382 nm but the highest absorbance was at 376 nm. So, this wave length was determined to be the maximum wave length for quantitative evaluation TC-HCl (Figure 4).

Figure S4: UV spectrum of tetracycline HCl on scanning in the wave-length range of 260-410 nm

### Forced degradation studies

Band impurities formed under stress conditions are observed separately under image visualizer 2 at 366 nm (Figure 5). As shown in Figure 6, the formed area of the peak decreases due to degradation of principal analyte with stress conditions. Hence, the developed method can be utilized for analysis of analyte in the presence of impurities formed during stress conditions.


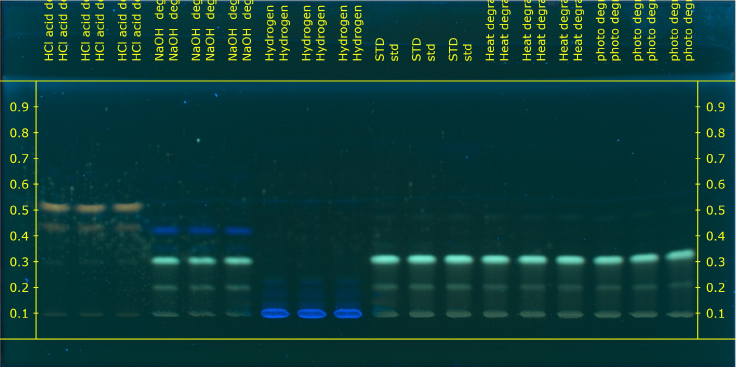

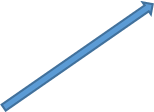


TC-HCl band

Figure S5: Bands of TC-HCl and products of its forced degradation with optimized mobile phase at 366 nm under TLC visualizer

| 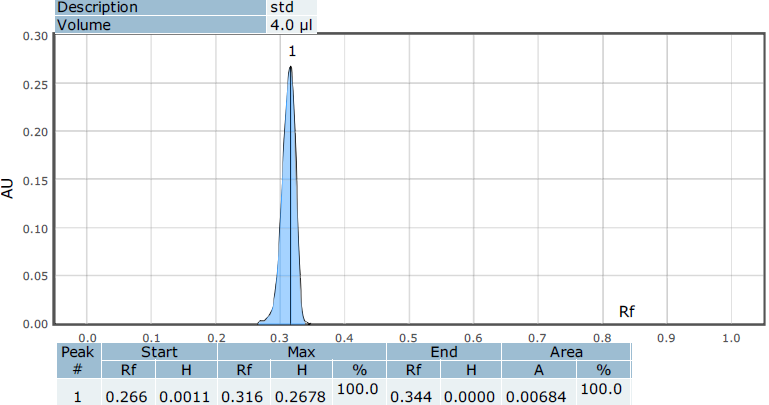 1 | 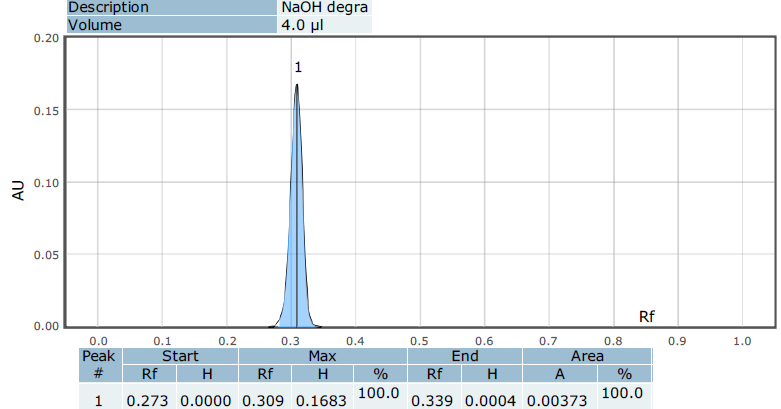 2 |
| --- | --- |
| 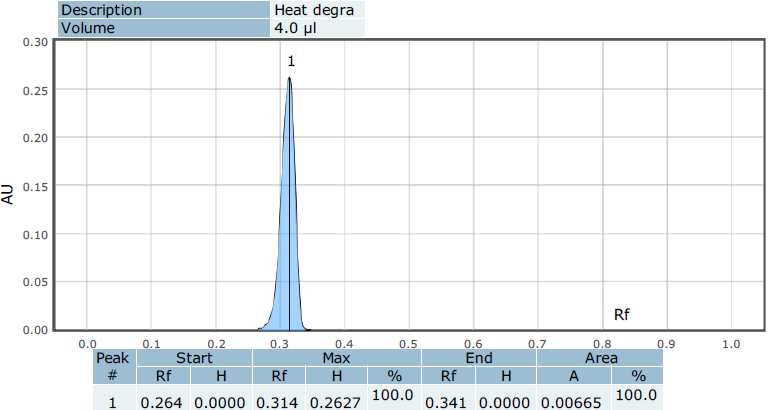 3 | 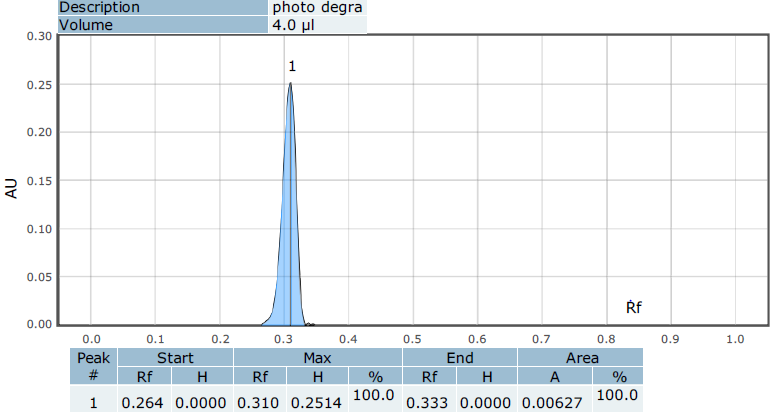 4 |

Figure S6: Peaks for standard TC-HCl (1) and TC-HCl under stress conditions (0.1M NaOH (2), heated (3) and exposed to 254 nm (4)) for forced degradation study

## Method validation

### Linearity study

Figure S7: Calibration curve of Tetracycline HCl using HPTLC method

### Robustness study

Table S4: Execution of the fractional factorial experimental design for mobile phase composition and its responses to study robustness of the proposed HPTLC method

|  |  | Factor 1 | Factor 2 | Factor 3 | Factor 4 | Response 1 | Response 2 |
| --- | --- | --- | --- | --- | --- | --- | --- |
| Std | Run | A(ml) | B(ml) | C(ml) | D(ml) | PA for 300ng | RF |
| 9 | 1 | 4.4 | 19.6 | 10 | 6 | 0.005498 | 0.27 |
| 12 | 2 | 4.4 | 19.6 | 10 | 6 | 0.005258 | 0.306 |
| 4 | 3 | 4.6 | 19.8 | 9.8 | 5.8 | 0.005274 | 0.27 |
| 10 | 4 | 4.4 | 19.6 | 10 | 6 | 0.005508 | 0.275 |
| 6 | 5 | 4.6 | 19.4 | 10.2 | 5.8 | 0.005484 | 0.279 |
| 1 | 6 | 4.2 | 19.4 | 9.8 | 5.8 | 0.005354 | 0.292 |
| 8 | 7 | 4.6 | 19.8 | 10.2 | 6.2 | 0.005278 | 0.27 |
| 2 | 8 | 4.6 | 19.4 | 9.8 | 6.2 | 0.00558 | 0.283 |
| 5 | 9 | 4.2 | 19.4 | 10.2 | 6.2 | 0.00553 | 0.35 |
| 11 | 10 | 4.4 | 19.6 | 10 | 6 | 0.005498 | 0.303 |
| 7 | 11 | 4.2 | 19.8 | 10.2 | 5.8 | 0.005686 | 0.29 |
| 3 | 12 | 4.2 | 19.8 | 9.8 | 6.2 | 0.005442 | 0.29 |

*A-ethyl acetate volume (ml), B-acetone volume (ml), C-methanol volume (ml), D-1% ammonia solution volume (ml, PA-peak area (AU)

The 3D response surface plots were created as a function of significant variables, with the other variable remaining constant. It helps to visualize the effects. Increasing acetone volume with decreasing ethyl acetate volume, increasing methanol volume with decreasing ethyl acetate volume, and decreasing ethyl acetate volume at any level of 1% ammonia solution all resulted in an increasing peak area for the current method (Figure 8 (1A-1C)). Rf value had decreased during acetone volume increment with ethyl acetate volume increment and ethyl acetate volume increment at any level of methanol volume. Ethyl acetate volume decreasment with 1% ammonia solution volume increment produced increasing Rf (Figure 8 (2A-2C)).

As shown in Figure 9, increasing mobile phase volume with decreasing ADC 2 activation time or increasing tank saturation time resulted in an increased peak area (Figure 9 (1A-1C)). Figure 9 (2A-2C) shows that Rf value increased during decreasing tank saturation time along with mobile phase volume decreasing, mobile phase volume decreased at any level of ADC 2 activation time, and solvent migration distance increased with decreasing mobile phase or vice versa. Figures 8 and 9 had shown that the colour blue, green, and red can represent low, medium, and high responses, respectively. The majority of the 3D surface plots were green, as shown in Figures 8 and 9, indicating that small deliberate changes in factors had not been complemented by a significant change in the developed method's responses.

Table S5: Execution of the fractional factorial experimental design for different factors and its responses to study robustness of the proposed HPTLC method

|  |  | Factor 1 | Factor 2 | Factor 3 | Factor 4 | Response 2 | Response 3 |
| --- | --- | --- | --- | --- | --- | --- | --- |
| Std | Run | A(ml) | B(minute) | C(minute) | D(cm) | PA of 300ng | RF |
| 8 | 1 | 37 | 25 | 12 | 7.2 | 0.00523 | 0.22 |
| 4 | 2 | 37 | 25 | 8 | 6.8 | 0.00515 | 0.27 |
| 5 | 3 | 33 | 15 | 12 | 7.2 | 0.00478 | 0.33 |
| 12 | 4 | 35 | 20 | 10 | 7 | 0.0055 | 0.33 |
| 2 | 5 | 37 | 15 | 8 | 7.2 | 0.00525 | 0.3 |
| 7 | 6 | 33 | 25 | 12 | 6.8 | 0.00537 | 0.28 |
| 6 | 7 | 37 | 15 | 12 | 6.8 | 0.00492 | 0.33 |
| 10 | 8 | 35 | 20 | 10 | 7 | 0.0048 | 0.26 |
| 1 | 9 | 33 | 15 | 8 | 6.8 | 0.00502 | 0.29 |
| 11 | 10 | 35 | 20 | 10 | 7 | 0.00523 | 0.3 |
| 9 | 11 | 35 | 20 | 10 | 7 | 0.00519 | 0.29 |
| 3 | 12 | 33 | 25 | 8 | 7.2 | 0.00497 | 0.28 |

*A-mobile phase volume, B-tank saturation time, C-tank activation time, D-solvent migration distance, PA-peak area, FFD-fractional factorial design

| 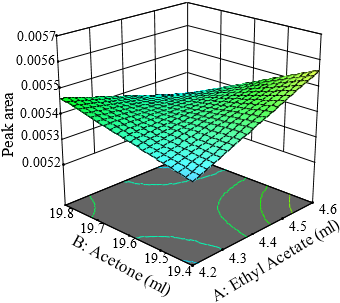 A | 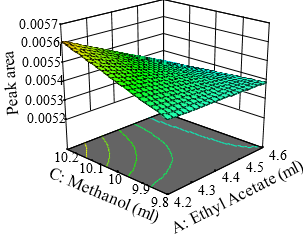 B | 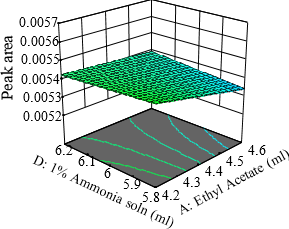 C |
| --- | --- | --- |
| 1. 3D of surface plot for MP composition change effect on peak area | | |
| 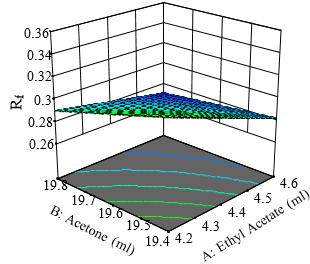A | 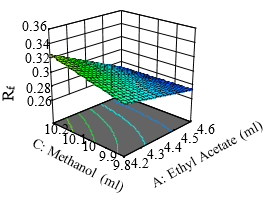B | 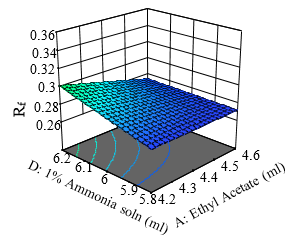C |
| 1. 3D of surface plot for MP composition change effect on peak area | | |

Figure S8: 3D surface plot showing MP composition change effect on response of HPTLC method (peak area (1) & R_f_ value (2)) tetracycline HCl

| 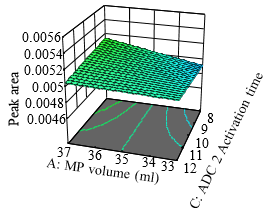 A | 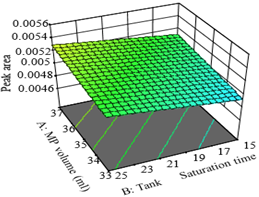 B | 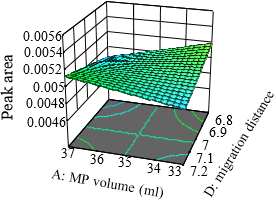 C |
| --- | --- | --- |
| 1. 3D of surface plot for different factors effect on peak area (PA) of HPTLC | | |
| 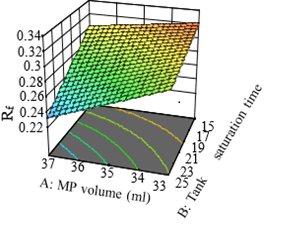 A | 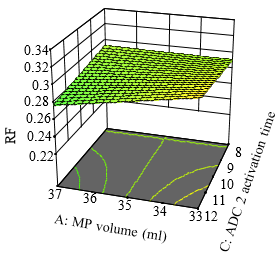 B | 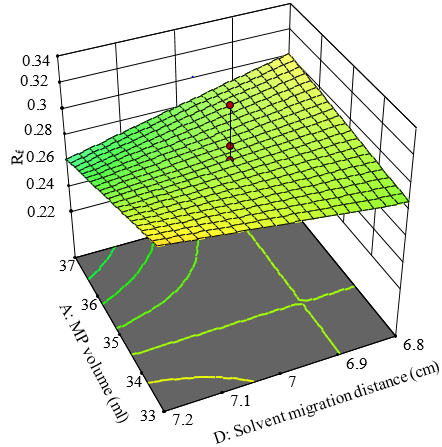 C |
| 1. 3D of surface plot for different factors effect on R_f_ of HPTLC | | |

Figure S9: 3D surface plot for effect of change of different factors on response of HPTLC method (peak area (1) & R_f_ (2))

Table S6: ANOVA analysis results for fractional factorial model of mobile phase composition change effect on HPTLC method responses

| Source | Peak Area | | | | | R_f_ | | | | | | |
| --- | --- | --- | --- | --- | --- | --- | --- | --- | --- | --- | --- | --- |
|  | SS | df | MS | F-value | p-value | SS | df | MS | | F-value | | p-value |
| Model | 1.50E-07 | 7 | 2.14E-08 | 1.91 | 0.278 | 4.60E-03 | 7 | 7.00E-04 | | 2.48 | | 0.1984 |
| A-Ethyl Acetate | 1.96E-08 | 1 | 1.96E-08 | 1.75 | 0.257 | 1.80E-03 | 1 | 1.80E-03 | | 6.85 | | 0.059 |
| B-Acetone | 8.98E-09 | 1 | 8.98E-09 | 0.7994 | 0.4218 | 9.00E-04 | 1 | 9.00E-04 | | 3.35 | | 0.141 |
| C-Methanol | 1.35E-08 | 1 | 1.35E-08 | 1.2 | 0.3353 | 4.00E-04 | 1 | 4.00E-04 | | 1.39 | | 0.3043 |
| D-1% Ammo soln | 1.28E-10 | 1 | 1.28E-10 | 0.0114 | 0.9201 | 5.00E-04 | 1 | 5.00E-04 | | 1.83 | | 0.2478 |
| AB | 7.14E-08 | 1 | 7.14E-08 | 6.36 | 0.0652 | 2.00E-04 | 1 | 2.00E-04 | | 0.7607 | | 0.4323 |
| AC | 3.28E-08 | 1 | 3.28E-08 | 2.92 | 0.1628 | 5.00E-04 | 1 | 5.00E-04 | | 1.83 | | 0.2478 |
| AD | 3.53E-09 | 1 | 3.53E-09 | 0.3141 | 0.6051 | 4.00E-04 | 1 | 4.00E-04 | | 1.39 | | 0.3043 |
| Residual | 4.49E-08 | 4 | 1.12E-08 |  |  | 1.10E-03 | 4 | 3.00E-04 | |  | |  |
| Lack of Fit | 4.51E-10 | 1 | 4.51E-10 | 0.0304 | 0.8727 | 0.00E+00 | 1 | 0.00E+00 | | 0.0307 | | 0.872 |
| Pure Error | 4.45E-08 | 3 | 1.48E-08 |  |  | 1.00E-03 | 3 | 3.00E-04 | |  | |  |
| Cor Total | 1.95E-07 | 11 |  |  |  | 5.60E-03 | 11 |  | |  | |  |
| Fit Statistics | | | | | | | | | | | | |
| Std. Dev. | 0.0001 | R² | | 0.7694 |  | Std. Dev. | 0.0162 | | R² | | 0.813 | |
| Mean | 0.0054 | Adjusted R² | | 0.3658 |  | Mean | 0.2898 | | Adjusted R² | | 0.4857 | |
| C.V. % | 1.94 | Predicted R² | | 0.2823 |  | C.V. % | 5.59 | | Predicted R² | | 0.414 | |
|  |  | Adeq Precision | | 4.7613 |  |  |  | | Adeq Precision | | 6.0426 | |
| Final Equation in Terms of Coded Factors | | | | | | | | | | | | |
| Peak Area=0.0054-0.0000A-0.0000B +0.0000C+4.000E-06D-0.0001AB-0.0001AC+0.0000AD | | | | | | RF=+0.2898 - 0.0150A - 0.0105B + 0.0067C + 0.0077D + 0.0050AB-0.0077AC—0.0067AD | | | | | | |

Table S7: ANOVA analysis results for fractional factorial model for different factors change effect on HPTLC responses

| mean peak area of 300ng | | | | | | Retention Factor (R_F_) | | | | |
| --- | --- | --- | --- | --- | --- | --- | --- | --- | --- | --- |
| Source | SS | df | MS | F-value | p-value | SS | df | MS | F-value | p-value |
| Model | 2.29E-07 | 3 | 7.64E-08 | 1.94 | 0.20 | 0.0087 | 6 | 0.0015 | 2.69 | 0.1489 |
| A-Mobile phase volume | | |  |  |  | 0.0005 | 1 | 0.0005 | 0.8333 | 0.4032 |
| B-Tanker Saturation time | 7.03E-08 | 1 | 7.03E-08 | 1.79 | 0.22 | 5.00E-03 | 1 | 5.00E-03 | 9.26 | 0.0287 |
| D-Solvent migration distance | | |  |  |  | 2.00E-04 | 1 | 2.00E-04 | 0.3704 | 0.5694 |
| AB |  |  |  |  |  | 8.00E-04 | 1 | 8.00E-04 | 1.48 | 0.2779 |
| AC | 2.10E-08 | 1 | 2.10E-08 | 0.53 | 0.49 | 4.00E-04 | 1 | 4.00E-04 | 0.8333 | 0.4032 |
| AD | 1.38E-07 | 1 | 1.38E-07 | 3.5 | 0.1 | 1.80E-03 | 1 | 1.80E-03 | 3.33 | 0.1275 |
| Residual | 3.15E-07 | 8 | 3.94E-08 |  |  | 2.70E-03 | 5 | 5.00E-04 |  |  |
| Lack of Fit | 6.57E-08 | 5 | 1.31E-08 | 0.158 | 0.96 | 2.00E-04 | 2 | 1.00E-04 | 0.12 | 0.891 |
| Pure Error | 2.49E-07 | 3 | 8.31E-08 |  |  | 2.50E-03 | 3 | 8.00E-04 |  |  |
| Cor Total | 5.44E-07 | 11 |  |  |  | 1.14E-02 | 11 |  |  |  |
| Fit Statistics | | | | | | | | | | |
| Std. Dev. | 2.00E-04 | R² | | 0.421 |  | Std. Dev. | 0.023 | R² | | 0.7632 |
| Mean | 5.10E-03 | Adjusted R² | | 0.2039 |  | Mean | 0.29 | Adjusted R² | | 0.4789 |
| C.V. % | 3.88 | Predicted R² | | 0.1069 |  | C.V. % | 8.01 | Predicted R² | | 0.4128 |
|  |  | Adeq Precision | | 4.8219 |  |  |  | Adeq Precision | | 6.1978 |
| Final Equation in Terms of Coded Factors | | | | | |  | | | | |
| PA=+0.0051+0.0001B-0.0001AC+0.0001AD | | | | | R_F_=+0.2900-0.0075A-0.0250B-0.0050D-0.0100AB-0.0075AC-0.0150AD | | | | | |

**P-values** less than 0.0500 indicate model terms are significant. In this case all factors except B-Tanker Saturation time change on R_F_ of HPTLC 2 are not a significant model term. The **Lack of Fit P-value** were greater than 0.05 for all models means that Lack of Fit is not significant relative. Non-significant lack of fit is good this mean the model to fit is achieved. For these models the difference between **Predicted R²** and Adjusted **R²** is less than 0.2 which is required to be like this. **Adeq Precision** measures the signal to noise ratio. A ratio greater than 4 is desirable. The ratios for all these models are greeter this required limit so these models could be used to navigate the design space. The equation in terms of coded factors can be used to make predictions about the response for given levels of each factor. By default, the high levels of the factors are coded as +1 and the low levels are coded as -1. The coded equation is useful for identifying the relative impact of the factors by comparing the factor coefficients.

### Analysis of bulk substance and commercial dosage forms HPTLC

| 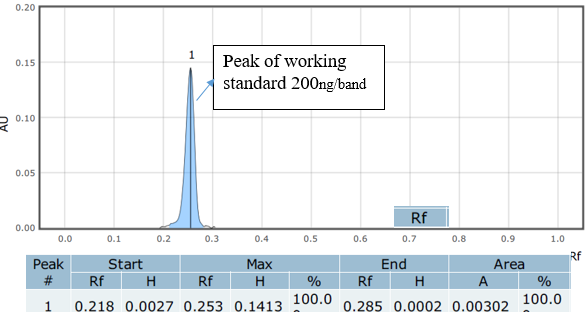A | 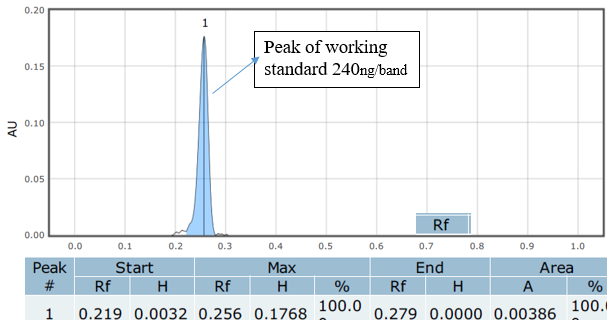B |
| --- | --- |
| 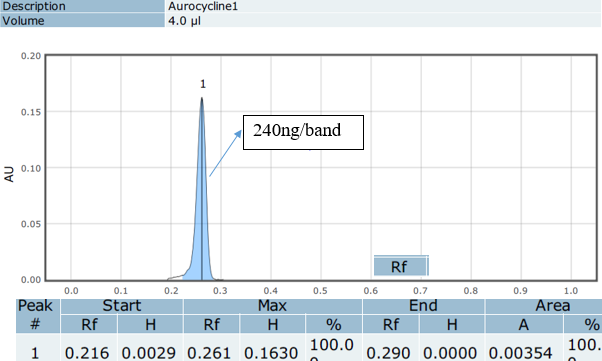C | 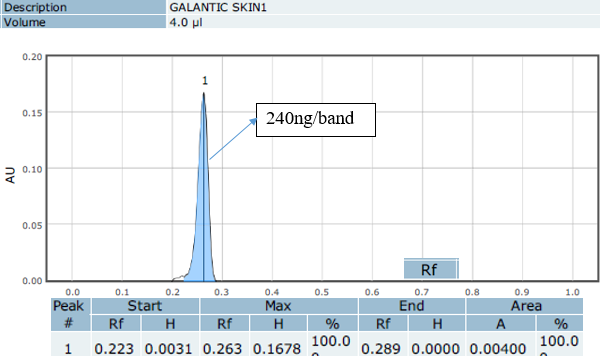 D |

Figure S10: Peak of tetracycline HCl API and eye ointment pharmaceutical products with HPTLC method

| 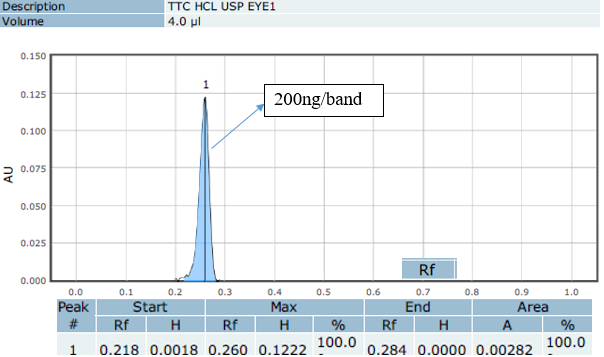A | 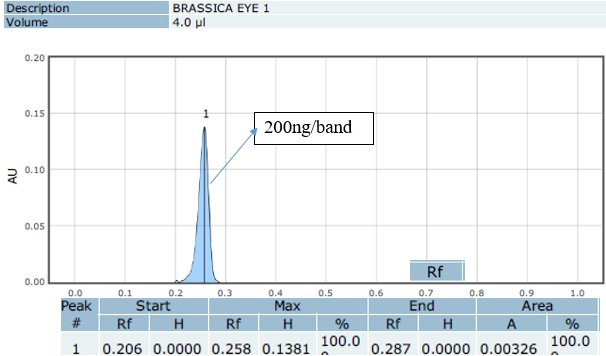 B |
| --- | --- |
| 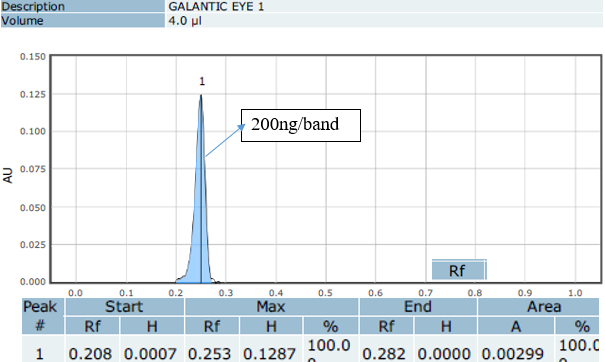 C | 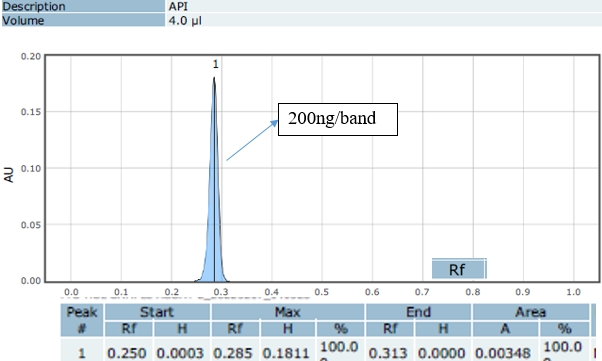 D |

Figure S11: Peak of TC-HCl working standard and skin ointment pharmaceutical products with HPTLC method

| 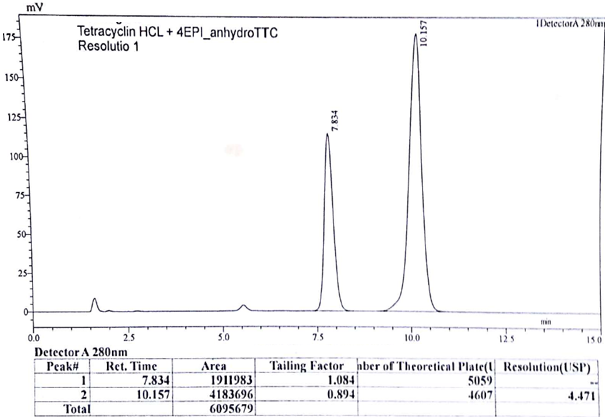 A |
| --- |
| 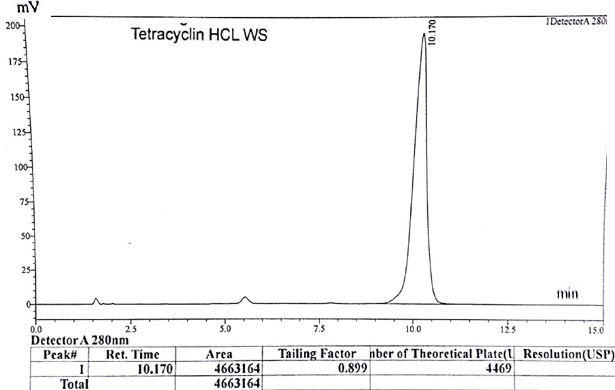 B |

### Assay of bulk substance and commercial dosage form of tetracycline HCl using HPLC

Figure S12: Peak of mixture of TC-HCl and 4-EATC (A) and TC- HCl WS using official HPLC method

| 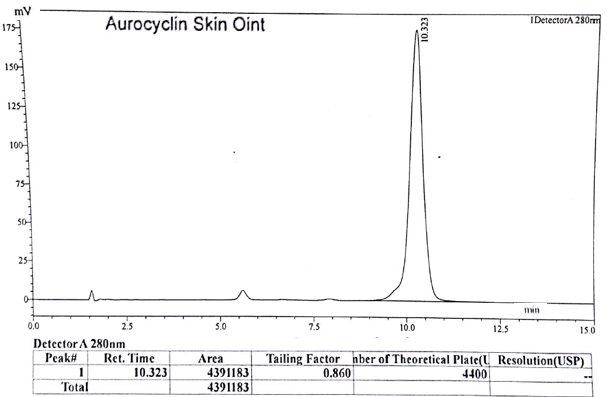A |
| --- |
| 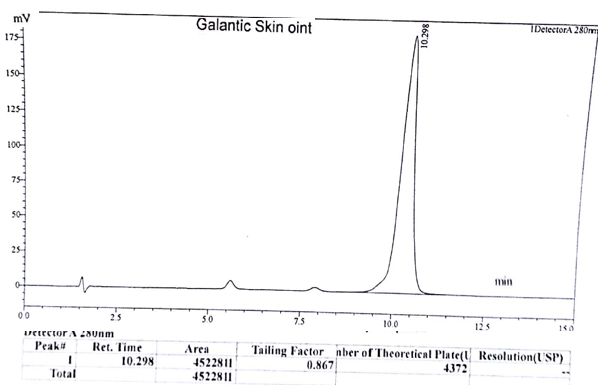B |

Figure S13: Peak of TC-HCl skin ointment with official HPLC method

| 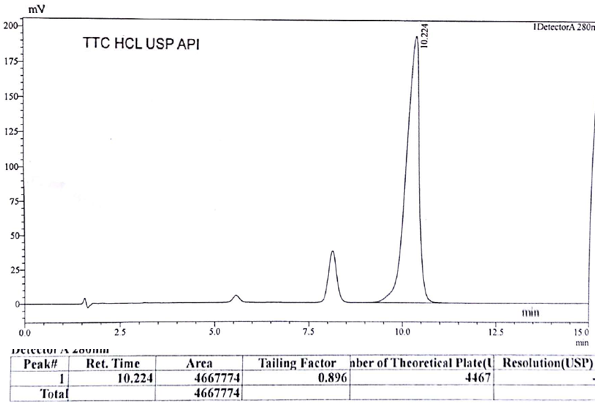 A |
| --- |
| 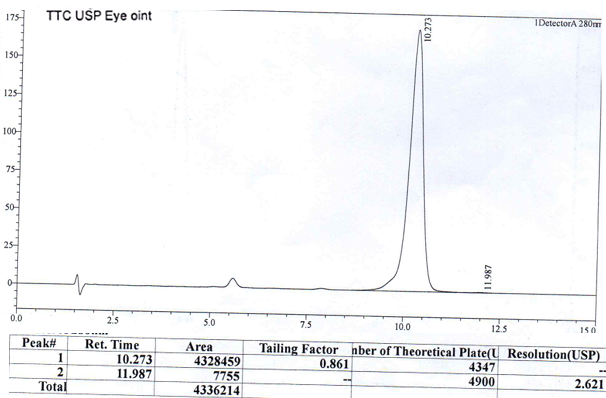 B |

Figure S14: Peak of TC-HCl API and eye ointment with official HPLC method

| 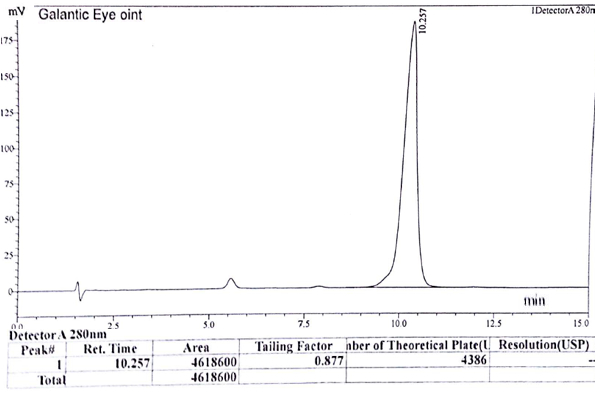 A |
| --- |
| 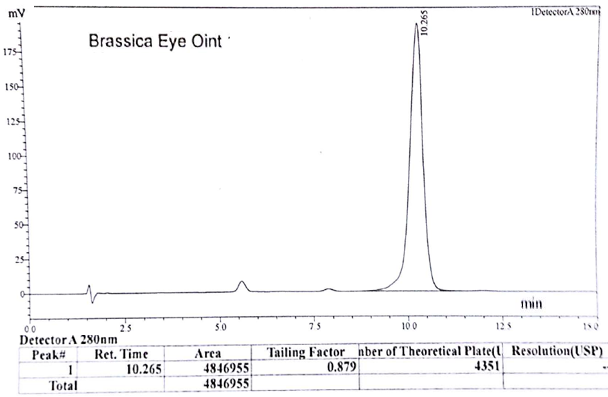B |

Figure S15: Peak of TC-HCl eye ointment with USP HPLC method

Transferability of the developed method for semi-automated HPTLC

####
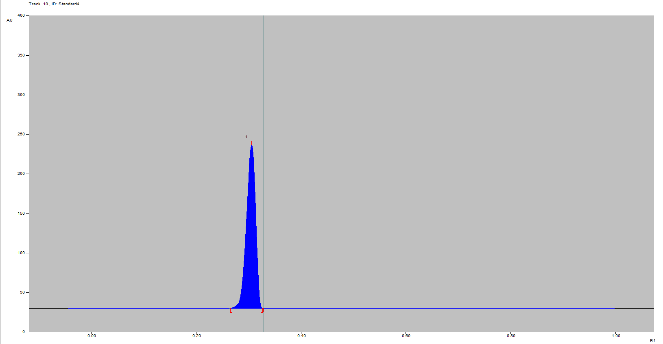

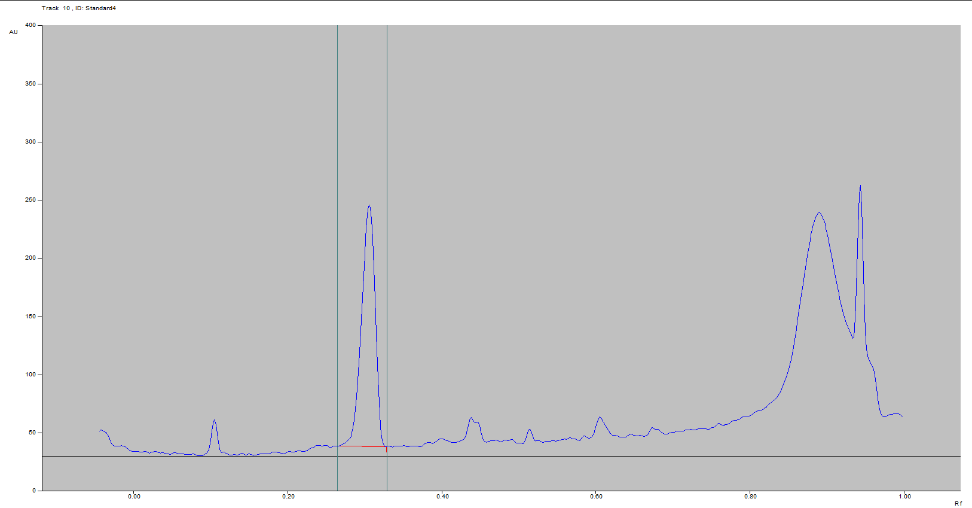
Rf of tetracycline HCl with semi-automated HPTLC

A B

Figure S16: Peak for tetracycline HCl (Rf, 0.30±0.02) with semi-automated HPTLC without background noise correction (A) and with background noise correction (B)

#### Method validation

##### Specificity


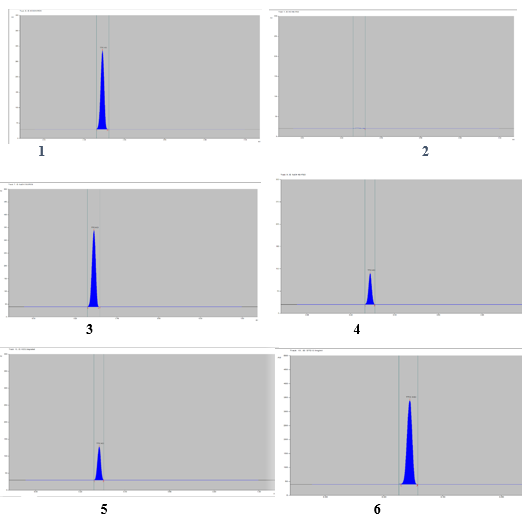


Figure S17: Degradation of tetracycline HCl in 0.1M HCl at environmental T^O^ (1), 0.1M HCl at 80^O^c for 2hrs (2), 0.1M NaOH at environmental T^O^ (3), 0.1M NaOH at 80^O^c for 2hrs (4), 3% H_2_O_2_ solution (5) and standard solution (6)

Table S8: Peak area of 400 ng/band TC-HCl in stress conditions using semi-automated HPTLC

| condition | formulation | Amount/con | Temperature and time | Peak area |
| --- | --- | --- | --- | --- |
| STD | Solution | 0.1mg/mL | Room Tº | 4853.7 |
| Uv chamber | powder | 50 mg | 254 nm light for 6hrs | 4421.73 |
| Oven | powder | 50 mg | 60 ºC for 6 hrs | 4343.6 |
| 3.0% H_2_O_2_ | solution | 0.1 mg/mL | Room Tº for 2 hrs | 769.25 |
| 0.1 M NaOH | solution | 0.1 mg/mL | 80 ºC for 2 hrs | 1258.42 |
| 0.1 M HCl | solution | 0.1 mg/mL | 80 °C for 2 hrs | 0 |

*con-concertation

##### Linearity and range

Figure S18: Calibration curve of tetracycline HCl for semi-automated HPTLC

Table S9: Calibration parameters obtained from the semi-automated HPTLC method.

| Parameter | Result |
| --- | --- |
| Calibration range (ng/band | 160-560 |
| Coefficient of x^2^ | -7*10^-9^ |
| Coefficient of x | 2*10 ^-5^ |
| Y-intercept | -0.0005 |
| Determination coefficient (R^2^) | 0.9999 |
| Correlation coefficient (r) | 0.99995 |
| polynomial regression equation | Y=-7*10^-8^ x^2^+2*10^-5^ x-0.0005 |

##### Accuracy

The percentage recovery of added amount sin the dosage form at 80 %, 100 %, and 120 % was 101.25 %, 97.0 % and 98.75 % respectively. The average recovery of the three levels was 99.0 %, confirming the accuracy of the developed method. Results of all levels averaged out to meet the percentage recovery acceptance criteria (98-102 %).

##### Precision

| ng/ band | Method precision | | | | | |
| --- | --- | --- | --- | --- | --- | --- |
|  | Intra-day precision | | | Intermediate precision | | |
|  | MPA | SD | %RSD | MPA | SD | %RSD |
| 160 | 2638 | 46 | 1.8 | 2660 | 32 | 1.2 |
| 200 | 3199 | 38 | 1.2 | 3222 | 30 | 0.9 |
| 240 | 3841 | 31 | 0.8 | 3873 | 26 | 0.7 |
| System precision | | | | | | |
|  | Sample application | | | Sample scanning | | |
| 200 | 2034.29 | 40.43 | 1.99 | 2274.37 | 35.53 | 1.56 |

Table S10: method precision (Repeatability and intermediate precision) (n=3) at three level and system precision of the developed method at 200 ng/band (n=6)

N.B.: MPA-mean peak area (n=3), SD-standard deviation, RSD-relative standard deviation

##### Robustness

| 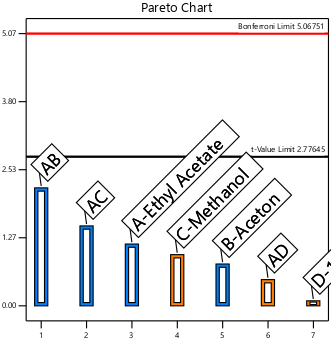 A | 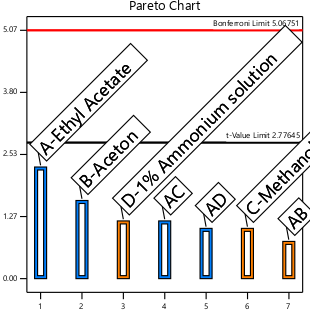 B |
| --- | --- |
| 1. Mobile phase composition change effect on peak area(A) and R_F_ (B) | |
| 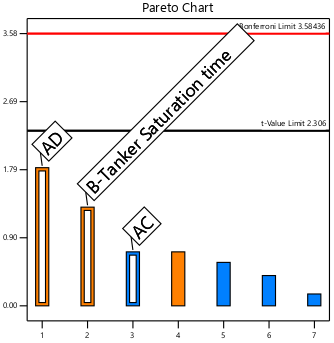 A | 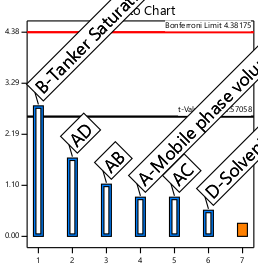 B |
| 1. Different factors change effect on peak area (A) and R_F_ (B) | |

Figure S19: Pareto charts of rank (X-axis) vs t-value of |effect| (Y-axis) showing the effect of MP composition change (1) and change of other factors (2) on responses of semi-automated HPTLC

|  |  | Factor 1 | Factor 2 | Factor 3 | Factor 4 | Response 1 | Response 2 |
| --- | --- | --- | --- | --- | --- | --- | --- |
| Std | Run | A:Ethyl | B:Aceton | C:Methanol | D:1% Ammo soln | PA 240ng | R_F_ |
|  |  | ml | ml | ml | ml | AU | __ |
| 2 | 1 | 4.2 | 19.4 | 10.2 | 6.2 | 2961.31 | 0.31 |
| 1 | 2 | 4.2 | 19.8 | 10.2 | 5.8 | 3153.19 | 0.3 |
| 6 | 3 | 4.4 | 19.6 | 10 | 6 | 3052.65 | 0.31 |
| 10 | 4 | 4.2 | 19.8 | 9.8 | 6.2 | 2714.51 | 0.32 |
| 7 | 5 | 4.2 | 19.4 | 9.8 | 5.8 | 2845.76 | 0.29 |
| 8 | 6 | 4.4 | 19.6 | 10 | 6 | 3027.23 | 0.3 |
| 9 | 7 | 4.6 | 19.8 | 10.2 | 6.2 | 2985.72 | 0.31 |
| 3 | 8 | 4.6 | 19.4 | 9.8 | 6.2 | 2872.54 | 0.3 |
| 12 | 9 | 4.4 | 19.6 | 10 | 6 | 3058.21 | 0.29 |
| 5 | 10 | 4.6 | 19.8 | 9.8 | 5.8 | 3127.23 | 0.31 |
| 11 | 11 | 4.6 | 19.4 | 10.2 | 5.8 | 2976.23 | 0.3 |
| 4 | 12 | 4.4 | 19.6 | 10 | 6 | 2793.39 | 0.31 |

Table S11: MP composition change effect on robustness of HPTLC-1 method with FFD

Where: Ethyl-ethyl acetate, Ammo Soln-ammonium solution, PA-peak area

Table S12: Testing of robustness of factors change for semi-automated HPTLC method full factorial design.

|  |  | Factor 1 | Factor 2 | Factor 3 | Response 1 | Response 3 |
| --- | --- | --- | --- | --- | --- | --- |
| Std | Run | A (ml) | B(minute) | C(cm) | PA of 300ng (AU) | mean Rf |
| 9 | 1 | 20 | 30 | 7 | 3622.67 | 0.3 |
| 5 | 2 | 18 | 25 | 7.2 | 3694.37 | 0.31 |
| 8 | 3 | 22 | 35 | 7.2 | 3645.9 | 0.3 |
| 7 | 4 | 18 | 35 | 7.2 | 3552.17 | 0.29 |
| 11 | 5 | 20 | 30 | 7 | 3670.1 | 0.29 |
| 6 | 6 | 22 | 25 | 7.2 | 3765 | 0.3 |
| 10 | 7 | 20 | 30 | 7 | 3757.1 | 0.31 |
| 1 | 8 | 18 | 25 | 6.8 | 3672.39 | 0.29 |
| 4 | 9 | 22 | 35 | 6.8 | 3849.67 | 0.31 |
| 2 | 10 | 22 | 25 | 6.8 | 3535.37 | 0.31 |
| 3 | 11 | 18 | 35 | 6.8 | 3653.3 | 0.3 |

Where: A-mobile phase volume, B-development champer saturation time, C-solvent migration distance, FD- factorial design

| 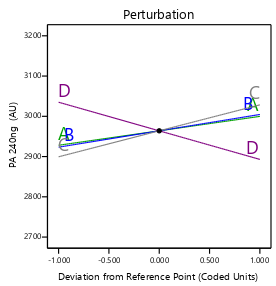 A | 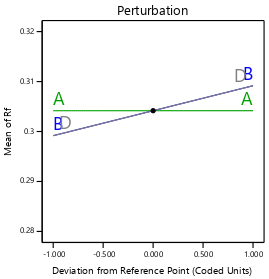 B |
| --- | --- |
| 1. Mobile phase composition change effect on Peak area (A) and RF (B)  Where: A-ethyl acetate, B-acetone, slope, C-methanol, and D- 1% aqueous ammonia | |
| 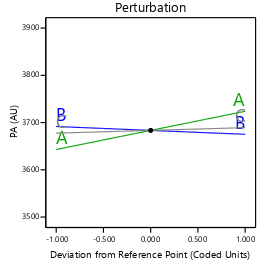 A | 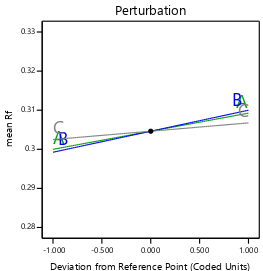 B |
| 1. Different factors change effect on Peak area (A) and RF (B)   Where: A-MP volume, B-saturation time and C- migration distance | |

Figure S20: Perturbation plot for peak area response (1A&2A) R_F_ response (1B&2B) for effect of factors on responses HPTLC-1 method.

Table S13: Statistical Parameters from ANOVA for semi-automated HPTLC

|  | parameters | 240ng/band (MP comp) | | 300ng/band (Diff factor effect) | |
| --- | --- | --- | --- | --- | --- |
|  |  | PA | R_F_ | PA | R_F_ |
|  | Model p-value | 0.1805 | 0.0672 | 0.1433 | 0.1252 |
|  | Lack of Fit | 0.9165 | 0.9940 | 0.8766 | 0.9721 |
|  | C.V. % | 3.42 | 2.12 | 1.53 | 2.11 |
|  | R-Squared | 0.7402 | 0.6729 | 0.8877 | 0.65133 |
|  | Adj R-Squared | 0.4284 | 0.4860 | 0..6631 | 0..4189 |
|  | Pred R-Squared | 0.4314 | 0.5822 | 0.6417 | 0.3130 |
|  | Adeq Precision | 5.4180 | 7.2000 | 6.3927 | 5.2668 |
|  | Equation | PA =  +29634  +35.87A  +40.60B  +64.55C  -71.04D  +25.44AB  -74.01AC | Rf =  +0.3042  +0A  +0.005B  +0.005D  -0.005AD | PA =  3671.02  +27.96A  -6.66C  +44.56AB  +13.13AC  -69.56BC  -38.79ABC | Rf =  +0.3009  +0.0038A’  -0.0038A’C’  -0.0038B’C’  +0.0038A’B’C’ |

Where: For mobile phase composition (A-ethyl acetate, B-acetone, C-methanol D-1% Ammo soln); for different factors (A’-MP volume B’- saturation time & C’- migration distance), PA- Peak area, R_F_= retention factor

| 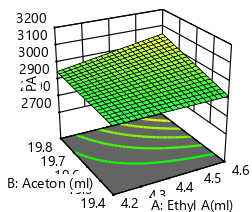 A | | 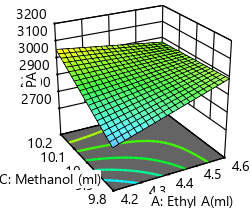 B | | |
| --- | --- | --- | --- | --- |
| 1. 3D of surface plot for MP composition variation effect on PA of HPTLC 1 | | | | |
| 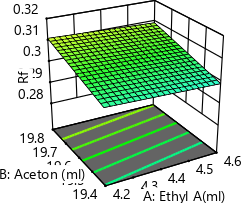 A | | 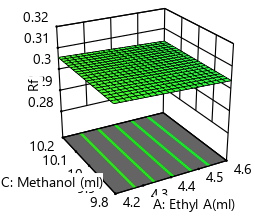 B | | |
| 1. 3D of surface plot for MP composition variation effect on R_F_ of HPTLC1 | | | | |
| 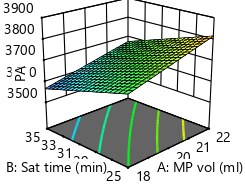 A | 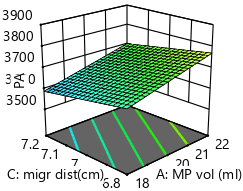 B | | 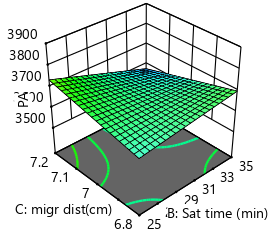 C | |
| 1. 3D of surface plot for different factors effect on peak area (PA) of HPTLC | | | | |
| 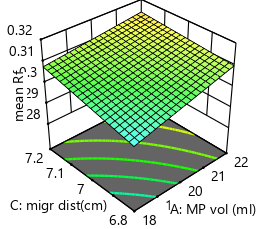 A | 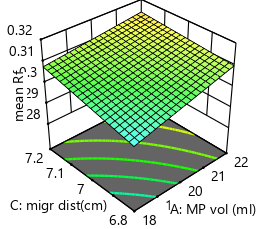 B | | | 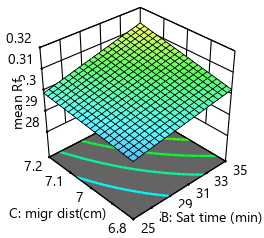 C |
| 1. 3D of surface plot for different factors effect on peak area (PA) of HPTLC-1 | | | | |
| Where: different factors; MP vol- mobile phase volume, migr dist- solvent migration distance, satur time-chamber saturation time. Active time-ADC 2 activation time | | | | |

Figure S21: 3D surface plot showing effect of MP composition and different factors on HPTLC-1 response (1and 3–peak area. And 2 and 4–R_F_-value)

##### Sample stability study

Table S14: Stability study of 200 ng/band sample solution with semi-automated HPTLC method

| Time of analysis | MPA | Reduction (%) |
| --- | --- | --- |
|  |  |  |
| 30 minute | 2271.367 | - |
| 1 hr | 2261.122 | -0.45105 |
| 4 hrs | 2209.746 | -2.71295 |
| 8 hrs | 2164.382 | -4.71016 |
| 24 hrs | 2023.095 | -10.9305 |
| 48 hrs | 1976.502 | -12.9818 |
| 3 days | 1541.316 | -32.1415 |
